# Alkyl and Aryl Derivatives Based on *p*-Coumaric Acid Modification and Inhibitory Action against *Leishmania braziliensis* and *Plasmodium falciparum*

**DOI:** 10.3390/molecules25143178

**Published:** 2020-07-11

**Authors:** Susiany P. Lopes, Lina M. Yepes, Yunierkis Pérez-Castillo, Sara M. Robledo, Damião P. de Sousa

**Affiliations:** 1 PostGraduation Program in Technological Development and Innovation in Medicines, Federal University of Paraíba, João Pessoa CEP 58051-970, Brazil; susiany.lopes@academico.ufpb.br; 2 PECET-Facultad de Medicina, Universidad de Antioquia, Medellín Calle 70 # 52-21, Colombia; lina.yepes@udea.edu.co (L.M.Y.); sara.robledo@udea.edu.co (S.M.R.); 3 Escuela de Ciencias Físicas y Matemáticas, Universidad de Las Américas, Quito 170504, Ecuador; yunierkis.perez@udla.edu.ec; 4 Department of Pharmaceutical Sciences, Federal University of Paraíba, João Pessoa CEP 58051-970, Brazil

**Keywords:** hydroxycinnamic acids, natural products, leishmanicidal activity, antiplasmodial activity, cytotoxicity, neglected diseases

## Abstract

In low-income populations, neglected diseases are the principal cause of mortality. Of these, leishmaniasis and malaria, being parasitic, protozoan infections, affect millions of people worldwide and are creating a public health problem. The present work evaluates the leishmanicidal and antiplasmodial action of a series of twelve *p*-coumaric acid derivatives. Of the tested derivatives, eight presented antiparasitic activities **1**–**3**, **8**–**12**. The hexyl *p*-coumarate derivative (**9**) (4.14 ± 0.55 μg/mL; selectivity index (SI) = 2.72) showed the highest leishmanicidal potency against the *Leishmania braziliensis* amastigote form. The results of the molecular docking study suggest that this compound inhibits aldehyde dehydrogenase (ALDH), mitogen-activated kinase protein (MPK4), and DNA topoisomerase 2 (TOP2), all of which are key enzymes in the development of *Leishmania braziliensis*. The data indicate that these enzymes interact via Van der Waals bonds, hydrophobic interactions, and hydrogen bonds with phenolic and aliphatic parts of this same compound. Of the other compounds analyzed, methyl *p*-coumarate (64.59 ± 2.89 μg/mL; IS = 0.1) demonstrated bioactivity against *Plasmodium falciparum*. The study reveals that esters presenting a *p*-coumarate substructure are promising for use in synthesis of derivatives with good antiparasitic profiles.

## 1. Introduction

Neglected diseases typically affect tropical and subtropical regions, principally developing countries [1,2], with poor and marginalized people [3] being the most affected. According to the World Health Organization, neglected diseases affect more than 1 billion people around the world [4]. These diseases cause great impacts on public health and include Chagas disease, leishmaniasis, malaria, schistosomiasis, onchocerciasis, lymphatic filariasis, trypanosomiasis and dengue [5].

Leishmaniasis, caused by parasitic protozoans of the *Leishmania genus*, is a neglected disease that affects both humans, and domestic and wild mammals [6,7,8,9,10]. These parasites: *L.* (*Viannia*) *braziliensis*, *L*. (*V.*) *guyanensis*, *L*. (*V.*) *lainsoni*, *L*. (*V*.) *shawi*, *L*. (*V*.) *naiffi*, *L*. (*V*.) *lindenbergi* and *L*. (*Leishmania*) *amazonensis* affect more than 98 countries worldwide [11,12]. Caused by unicellular eukaryotic protozoans of the *Plasmodium* genus such as *Plasmodium falciparum* and *Plasmodium vivax*, leishmaniasis quickly develops into a very serious human infection that can cause death [13,14,15]. Approximately 12 million people are currently affected with the disease [16]. Malaria however, remains the most prevalent parasitic infection in the whole world with more than 3.4 billion people infected, and roughly 1.2 billion cases per year [17,18,19]. Chloroquine is the current drug of choice for treatment of malaria. However, due to the development of drug resistance, it has become necessary to investigate new drug candidates [20,21,22,23].

Hydroxycinnamic acids are natural phenolic compounds found in fruits, cereals, and coffee [24,25]. Caffeic acid, *p*-coumaric acid and ferulic acid are principal representatives of this class [26,27,28,29,30,31,32]. Various pharmacological activities for *p*-coumaric acid have been noted, stimulating many studies using both in vivo (animal) and in vitro models to investigate this compound and its synthetic derivatives [33,34,35,36]. In addition to its applicability in the food, cosmetics, chemical, and pharmaceutical products, reports of its antioxidant, antimicrobial, anti-inflammatory, antineoplastic, antimalarial, anti-Alzheimer’s and neuroprotective effects are abundant [37,38,39,40]. *p*-Coumaric acid presents antiparasitic activity against *L. amazonensis* [41]. A derivative of *p*-coumaric acid, methyl *p*-coumarate, presents activity against *P. falciparum* [42]. There are also reports regarding *p*-coumaric acid and inhibition of *P. falciparum* dihydropteroate synthase [43]. The aim of the study was to prepare *p*-coumaric acid derivatives and evaluate their antiprotozoal activity. To deduce possible sites of antiparasitic action, in silico modeling was also performed.

## 2. Results and Discussion

### 2.1. Chemistry of the Compounds 1–12

**Figure 1:**
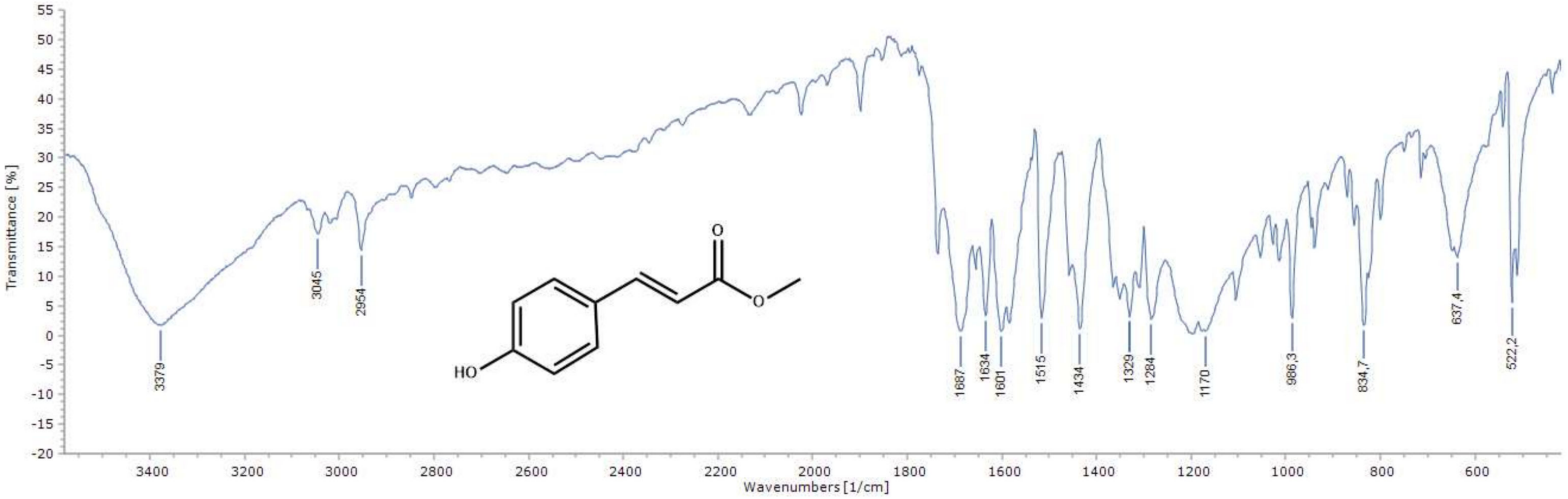
Infrared spectrum (cm^−1^) of methyl *p*-coumarate (**1**).

**Scheme 1:**
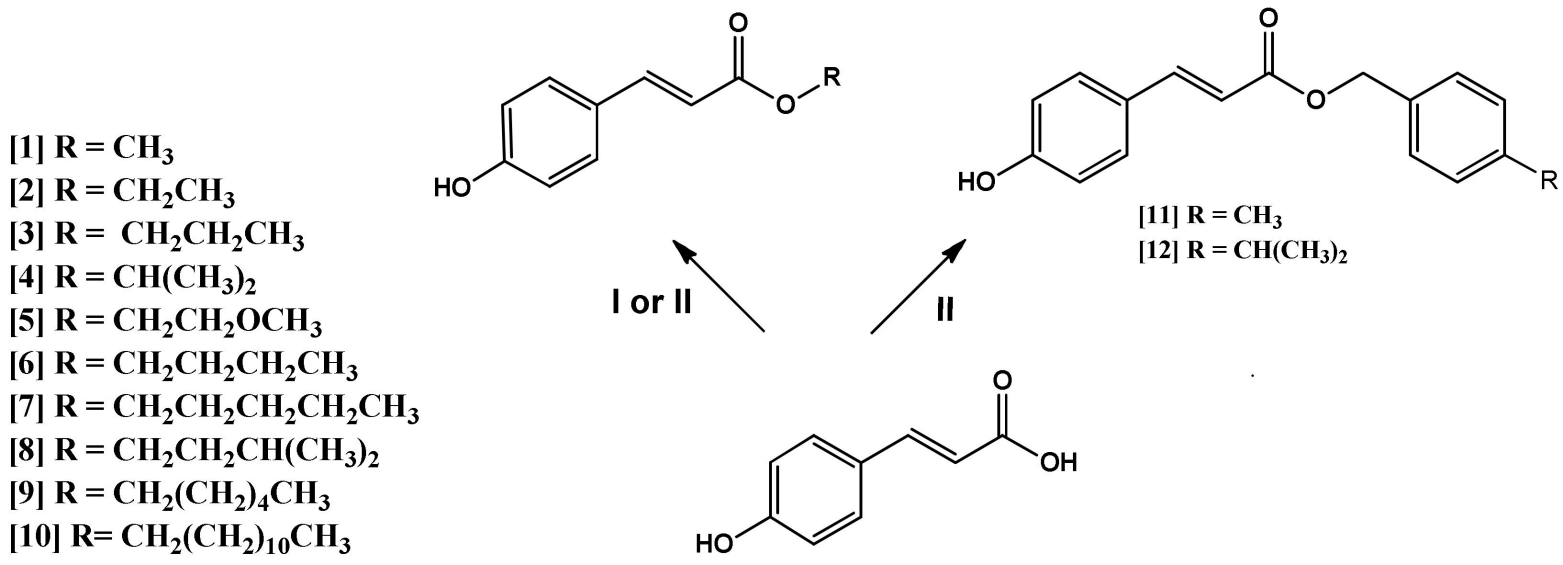
Preparation of the *p*-coumaric acid derivatives: (**I**) alcohol, H_2_SO_4_, reflux; (**II**) alcohol, THF, TPP, DIAD, 0 °C to room temperature.

In this study, twelve compounds (Scheme 1), containing the (E)-3-(4-hydroxyphenyl) acrylic acid substructure were synthesized; exclusively changing the radical R to methyl (Me (**1**)), ethyl (Et (**2**)), propyl (*n*-Pr (**3**)), isopropyl (*i*-Pr (**4**)), methoxyethyl (MeOEt (**5**)), butyl (Bu (**6**)), pentyl (*n*-Pent (**7**)), isopentyl (*i*-pent(**8**)), hexyl (Hex (**9**)), dodecyl (Dodec (**10**)), 4-methylbenzyl (MBz (**11**)), and 4-isopropylbenzyl (*i*-PrBz (**12**)) esters. Acrylic acid and alcohol were used in the reactions with (E)-3-(4-hydroxyphenyl) (Scheme 1). Products were prepared by Fischer esterification and Mitsunobu reaction (Scheme 1). The compounds were characterized by infrared spectra (Figure 1) and Nuclear Magnetic Resonance (NMR) spectra, as evidently published by Lopes et al. [44].

### 2.2. Antileishmanial and Antiplasmodial Activity of Compounds 1–12

The results were evaluated using the 50% effective concentration (EC_50_) for parasitic activity. We analyzed the collection (twelve compounds) for activity against *Leishmania braziliensis* in its amastigote form, against *Plasmodium falciparum*, and for cytotoxicity against U-937 macrophages using 50% lethal concentration (LC_50_). Of the derivatives analyzed in Table 1, eight compounds (**1**–**3**, and **8**–**12**) showed moderate to good activity against *Leishmania braziliensis* amastigotes. However, the twelve compounds exhibited weak antiplasmodial activity.

### 2.3. Antileishmanial Activity

The simplest structure, (compound **1**) methyl *p*-coumarate demonstrated excellent leishmanicidal activity (EC_50_ of 8.28 ± 0.14 μg/mL). Substitution with an ethyl group (compound **2**), potentiated the inhibitory effect and a decrease in EC_50_ (4.91 ± 0.64 μg/mL) was observed when compared to compound **1**. Increases in side chain length help to increase lipophilicity and consequently decrease EC_50_ values. These results are in agreement with a study by Nóbrega et al. (2019) [45], demonstrating that ethyl 3,4,5-trimethoxycinnamate presented better leishmanicidal activity than methyl 3,4,5-trimethoxycinnamate. However, Lima et al. (2016) [46] demonstrated that ethyl caffeate presents no activity against *Leishmania amazonensis*. In contrast, excellent cytotoxic activity was observed against U-937 macrophage cells for both compounds **1** and **2** (respectively 6.26 ± 0.03 μg/mL and 1.06 ± 1.08 μg/mL).

Compounds presenting three side chain carbons (**3**, **4**, and **5**) exhibited differing EC_50_ values (respectively 6.3 ± 1.14 μg/mL, 28.33 ± 0.27 μg/mL, and 86.98 ± 18.13 μg/mL). This may be due to an alteration in the n-propyl radical (**3**) to isopropyl (**4**), contributing to the decrease in leishmanicidal activity seen in further branching in compound **4** [47,48]. Compound **3** was fourteen times more active than compound **5**. The presence of the oxygen atom in the compound **5** side chain likely contributes to a reduction in antiparasitic activity. Meanwhile, Nóbrega et al. (2019) [45] observed that the presence of side chain oxygen in a methoxy-ethyl derivative (3,4,5-trimethoxycinnamate) increases leishmanicidal activity. The leishmanicidal activity values of both compounds **4** and **5** were lower (more potent) than their cytotoxic activity values against U-937 macrophage cells (respectively 96.23 ± 18.89 μg/mL, 126.71 ± 16.5 μg/mL. These same cytotoxicity values were also much higher (less potent) than that of compound **3** (11.89 ± 0.8 μg/mL) which had high cytotoxicity.

Butyl *p*-coumarate (**6**) being compared to compound **1**, presented the lowest leishmanicidal potency, with an EC_50_ of 323.29 ± 50.04 μg/mL. Interestingly, Bernal et al. (2019) [49] found that groups with a medium length linear alkyl chain exhibited higher activity against *L. donovani* when compared to groups presenting short chains [50]. Meanwhile, pentyl *p*-coumarate (**7**) was five times less potent than isopentyl *p*-coumarate (**8**) (32.03 ± 5.054.3 μg/mL, 6.02 ± 0.22 μg/mL, respectively), possibly due to compound **8** branching. When compared as to cytotoxicity, neither expressed a significant difference (respectively 16.09 ± 3.81 μg/mL and 19.58 ± 1.76 μg/mL). Steverding et al. (2016) [51] also observed antiparasitic activity for isopentyl caffeate against *Trypanosoma brucei*.

Hexyl *p*-coumarate (**9**), dodecyl *p*-coumarate (**10**), 4-methylbenzyl *p*-coumarate (**11**) and 4-isopropylbenzyl *p*-coumarate (**12**), (respectively 4.14 ± 0.55 μg/mL, 16.34 ± 0.49 μg/mL, 5.69 ± 0.3 μg/mL and 11.21 ± 0.2 μg/mL) displayed high leishmanicidal activity, likely due to their relatively high lipophilicity. Yet Otero et al. (2014) [52] found in their study that hexyl cinnamate presents less leishmanicidal activity than dodecyl cinnamate. However, in the present study **9** was considered the most potent compound compared to both compound **10** and the other derivatives analyzed. The results obtained are in accordance with the preparation plan for the compounds, in which alkyl and benzyl substituents were added to increase the lipophilicity of the compounds. In this way, compounds with good absorption were obtained to cross the parasite membrane resulting in greater bioavailability [53,54,55]. Several mechanisms of action can occur, such as greater access to organelles that are biological targets for leishmanicidal activity [56]. As suggested in the molecular docking study, hexyl *p*-coumarate should inhibit the enzymes ALDH, MPK4, and TOP2 (see Table 2) which are essential for *Leishmania braziliensis* growth. Regarding the substitution of the aliphatic chain in the para position of the aromatic ring in compounds **11** and **12**, a difference in leishmanicidal activity was noted since compound **11** was twice as potent as compound **12** which possessed the more bulky substituent. These same results were also observed by Lopes et al. (2019) [44] in relation to trypanocidal activity.

### 2.4. Antiplasmodial Activity

Methyl *p*-coumarate (compound **1**) exhibited the lowest EC_50_ (64.59 ± 2.89 μg/mL) against *P. falciparum*. An increase in the side chain resulted in a decrease in antiplasmodial activity, since ethyl *p*-coumarate showed a higher EC_50_ (110.31 ± 30.84 μg/mL). Alson et al. (2018) [57] observed in their study that methyl caffeate and ethyl caffeate expressed the best antiplasmodial activities against *P. falciparum*. On the other hand, for Arsianti et al. (2018) [58], propyl *p*-coumarate (compound **3**) had low antiplasmodial activity when compared to isopropyl *p*-coumarate (**4**) and methoxy-ethyl *p*-coumarate (**5**), (respectively 139.04 ± 4.07 μg/mL, 106.08 ± 9.97 μg/mL, 98.66 ± 17.7 μg/mL). Furthermore, when comparing compound **1** with butyl *p*-coumarate (**6**), an increase in EC_50_ of 198.84 ± 7.9 μg/mL was observed.

If the side chain of the *p*-coumarate derivative is increased, an increase in the lipophilicity of the compounds is observed for pentyl *p*-coumarate of (**7**), isopentyl *p*-coumarate of (**8**), hexyl *p*-coumarate of (**9**) and dodecyl *p*-coumarate (**10**). As for antiplasmodial activity, there was a difference in EC_50_ between these compounds; compound 8 (95.48 ± 1.84 μg/mL) was more potent than compound **7** (156.37 ± 8.2 μg/mL). Yet Arsianti et al. (2017) [59] observed that alkyl ester groups presenting linear chains such as pentyl gallate, present better inhibitory action in relation to antiplasmodial activity than compounds with branched structures. When comparing compounds **9** and 10, there was a significant difference (respectively 206.35 ± 46.4 μg/mL, 81.19 ± 8.54 μg/mL); and hexyl *p*-coumarate showed the least antiplasmodial activity against *P. falciparum* [60]. In compounds **11** and **12** the substituent is a substituted aromatic ring; the antiplasmodial potency of these compounds was greater than the majority of the derivatives with carbon chains; both 4-methylbenzyl *p*-coumarate (**11**) and 4-isopropylbenzyl *p*-coumarate (**12**) presented weak antiplasmodial activity with respective EC_50_ of 83.98 ± 15.34 μg/mL and 93.66 ± 14.17 μg/mL.

### 2.5. Computational Methods

Computational target fishing approaches rely on the similarity principle: if a query compound is similar to a reference which binds a certain receptor, then the query might also bind the same receptor. Reference ligand–receptor interactions are generally extracted from large publicly available databases. However, the information in these databases is biased against human receptor–ligand associations. For this reason, the homology-based target fishing approach described in the Methods section was implemented to identify potential *Leishmania braziliensis* targets for compound **9**. The 16 identified potential *Leishmania braziliensis* targets for compound **9** are provided in Table 2. Of them, seven are annotated in the UniProt database as confirmed or predicted protein kinases, suggesting a subset of possible targets enriched with this function.

Molecular docking of compound **9** (to the 16 potential targets listed in Table 2) was thus performed as described in the Methods section. It has been shown that GSK3 inhibitors are effective in binding to ATP and substrate binding sites in both *Leishmania* spp. and in the human homolog [61,62]. Based on these findings, we explored GSK3 for ligand binding to these two sites in *Leishmania braziliensis*. TOP2 is an ATP-dependent DNA topoisomerase enzyme that contains ATP and nucleotide binding sites [63,64]. For the TOP2 docking calculations, only the ATP binding cavity was explored. The results of the consensus docking protocol are summarized in Table 3. Ten (10) of the 16 modeled targets, (for compound **9**), predicted more than one possible binding mode, leading to 33 ligand–receptor complexes needing further analysis Visual inspection of these 33 predicted complexes revealed meaningful binding modes for all of them. In all cases, the ligand was inside the receptor binding site, and either interaction or blocking of functionally relevant residues was noted.

Considering the same consensus protocol employed for ligand pose selection, the best docking results in GSK3 targets were obtained for the HDA2 and the ATP binding sites. However, the worst docking performance for GSK3 targets was observed for the PGFS and substrate binding sites. Interestingly, the GSK3 docking results presented large differences in scores for the ATP and substrate binding sites, which could be an indicator of selectivity towards ATP binding. Overall, the docking results did not allow for confident selection of the most probable compound **9** targets. Post-processing of predicted docking complexes using Molecular Dynamics (MD) simulations along with estimations of free energies of binding can help refine target prioritization [44,65]. Calculations were thus performed (described in the Methods section) to estimate the free energies of binding for compound **9** to its predicted targets using the MD simulations data. These simulation results are demonstrated in Table 4.

The MM-PBSA calculations reveal that due to the predicted positive free energies of binding, the complexes formed by compound **9** with HDA1, PKAC3, PKAC2, and GSK3 (SBS) are unfeasible. Though negative, the ΔGs of binding predicted for AKR, HDA2, PGFS, PGFS2, and YARS1, range from −1.08 kcal/mol to −2.01 kcal/mol. The values predict low stability for these complexes. Slightly better free energies of binding are predicted for the ALDH2, GSK3, CBPKC, CRK7, and PKC targets. Despite stable binding, (ΔG TOTAL < 0) is predicted for most of the predicted compound **9** targets, as seen in the results in Table 4, where the most probable compound **9** targets are ALDH, MPK4, and TOP2. One interesting result is that of the predicted likely compound **9** targets, the binding sites of TOP2 and the kinases (MPK4, PKC, GSK3, CBPKC, and CRK7) are all ATP binding cavities. This suggests that compound **9** might interfere with ATP binding in various proteins related to diverse cellular functions. Based on the results thus obtained, we selected ALDH, MPK4, and TOP2 as targets for additional evaluation concerning compound **9** potential anti-leishmanial mechanism of action.

**Figure 2:**
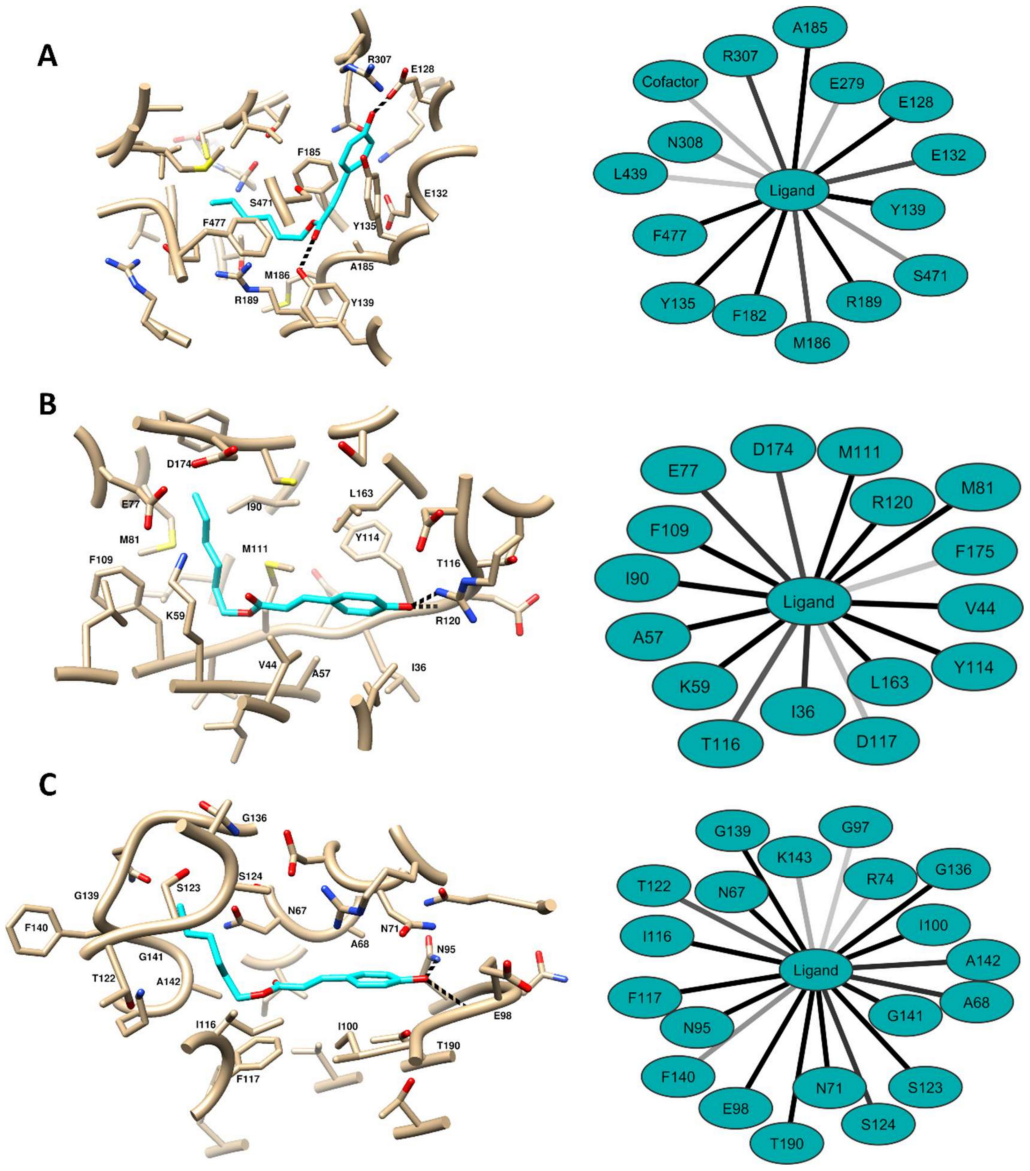
Predicted binding modes of compound **9** to its more probable receptors: (**A**) ALDH, (**B**) MPK4 and (**C**) TOP2. Receptors are depicted gray and compound **9** cyan. Non-carbon ligand and receptors atoms are colored following the scheme: red for oxygen, blue for nitrogen and yellow sulfur. Only receptors residues interacting with the ligand in more than 50% of the selected MD snapshots are labelled in the complexes. Hydrogen bonds are represented as black dashed lines. The observed networks of interactions along the MD snapshots used for MM-PBSA calculations are presented at the left. Darker lines in the interaction networks indicate more frequent interactions.

The interactions between compound **9** and its most probable targets were analyzed in detail along with the 200 snapshots employed for Molecular Mechanics with Poisson–Boltzmann and Surface Area solvation (MM-PBSA) calculations. The binding modes of the ligand to these receptors as predicted are shown in Figure 2. To choose a representative binding mode for each receptor, the 200 conformations of compound **9** were clustered and the centroid mode from the most populated cluster was selected. Figure 2 includes the network of interactions observed during the MD simulations between the ligand and the investigated proteins. Only the interactions taking place in more than 30% of the MD snapshots used for free energy of binding estimation are included in the figure. Given that our structural analyses are preformed over an ensemble of 200 conformations for each complex, not all hydrogen bond interactions are present in all snapshots. For this reason, only the hydrogen bonds observed in the selected representative structure of each complex are depicted as black dashed lines in Figure 2.

In the case of ALDH, the ligand binds to the substrate binding channel making contact with E128, E132, Y135, Y139, F182, A185, M186, R189, R307, S471 and F477 in more than 50% of the analyzed MD snapshots. As shown in Figure 2A, the hexyl tail occupies a sub-cavity close to the NAD cofactor, and hence completely blocks access, leading to enzyme blockage. In this predicted complex, the hydroxyphenyl ring of the compound interacts with residues at the entrance of the binding pocket. Most of the interactions observed were Van der Waals and hydrophobic. One factor adding stability to this complex is the presence of hydrogen bonds between the ligand’s hydroxyl moiety and E128 of ALDH. Compound **9** is also predicted to accept hydrogen bonds from the side chain of Y139 and the backbone of R189 through its carbonyl group. It is noteworthy that the hydrophobic hexyl fragment of the ligand localizes in a region containing hydrogen bond accepting capable residues such as T256, E279, T312, and the NAD cofactor. We postulate that inclusion of hydrogen bond donor substitutions in this part of compound **9** would improve the stability of this ALDH complex.

In the complex predicted for compound **9** with MPK4 (Figure 2B), interactions with I36, V44, A57, K59, E77, M81, I90, F109, M111, Y114, T116, A120, R120, L163 and D174 occur in the majority of the MD snapshots analyzed. The interactions are mainly Van der Waals and electrostatic in nature, and as in the complex predicted with ALDH, the ligand’s hexyl moiety orients toward the bottom of the cavity and the hydroxyphenyl substituent occupies the entrance of the binding site. In most of the MD snapshots studied, the hydroxyl group of the latter is predicted to achieve hydrogen bonding with either the backbone of Y114 or with side chains of R120 and D117. Another similarity between its predicted binding pose and that of ALDH is that the hydrophobic tail of compound 9 fits closely to two hydrogen bond acceptor amino acids at the receptor: E77 and D174.

The compound **9** complex predicted for the TOP2 ATP binding site (Figure 2C) reveals a large network contacts that includes N67, A68, N71, N95, E98, I100, I116, F117, T122, S123, S124, G136, G139, F140, G141, A142 and T190. Similar to its ALDH and MPK4 targets, the compound poses in TOP2 with its hexyl part oriented towards the bottom of the binding cavity and its aromatic substituent oriented towards the cavity entrance. The phenyl substituent is also capable of forming hydrogen bonds at the drug receptor site in the TOP2 complex, either through backbones E98 and T190 or via side chains of Q79 and N95. The cavity region where the aliphatic tail of compound **9** binds contains hydrogen bonding capable residues that include N67, S123, S124 and N138. This suggests that addition of hydrogen bond donor groups to the hydrophobic tail of the compound might increase its multitarget affinity against *Leishmania braziliensis* through ALDH, MPK4, and the TOP2–ATP binding site.

Of the three *Leishmania braziliensis* targets selected as the most likely for compound **9**, the aldehyde dehydrogenase function (ALDH) has been proposed as a process which (through inhibition) could be useful for development of therapeutic agents against *Leishmania* spp. [66]. Furthermore, there are several reports in the literature demonstrating the importance of MPK4 to *Leishmania* spp. in both development and infectious capability, making it an attractive for drug discovery efforts [67,68,69]. Finally, the importance of DNA topoisomerases in the life cycle of this type of parasite, and their use as drug targets has already been established in the scientific literature [70].

## 3. Conclusions

Twelve compounds were evaluated for antiparasitic potential against *L. braziliensis* amastigotes, against *Plasmodium falciparum*, and for cytotoxicity in U-937 macrophages. Eight of the derivatives were bioactive against *Leishmania braziliensis*, such as hexyl *p*-coumarate (compound **9**) (4.14 ± 0.55 μg/mL; IS = 2.72) which indicated the most potent inhibitory effect. The studies performed using molecular docking suggested that compound **9** interacts with the enzymes ALDH, MPK4, and TOP2, which are essential to *Leishmania braziliensis* development. Some of the compounds tested were bioactive against *P. falciparum* yet demonstrated little antiparasitic activity. These results may be useful to other studies involving *p*-coumaric acid, to help obtain ever more potent and selective derivatives against these protozoan species, which continue to compromise the quality of life of various world populations.

## 4. Materials and Methods

### 4.1. Reagents and Chemical Characterization 

The derivatives were purified by column adsorption chromatography (CC) using silica gel 60, ART 7734—MERCK, St. Louis, Missouri, EUA. Infrared spectra were performed using FTIR spectrophotometry and ^1^H and ^13^C NMR spectra were obtained using a Bruker-AscendTM machine, Bruker, Bremen, Germany, operating at 400 and 100 MHz. All compounds were structurally characterized as published by Lopes et al., 2019 [44].

### 4.2. General Procedure for Preparation of Compounds 1–8

*p*-Coumaric acid (0.1 g; 0.61 mmol) in alcohol (20 mL) was dissolved in the presence of H_2_SO_4_ (0.2 mL) and refluxed for complete reaction (5–27 h), being observed using single spot thin-layer chromatography (TLC) [71], as published by Lopes et al. [44].

### 4.3. Preparation of Compounds 9–12 by Mitsunobu Reaction

*p*-Coumaric acid (0.1 g; 0.61 mmol) and alcohol (0.61 mmol) were dissolved in 2.25 mL of tetrahydrofuran. The reaction mixture was stirred under magnetic stirring at 0 °C for 30 min. Subsequently, diisopropyl azodicarboxylate (0.12 mL; 0.61 mmol) and triphenylphosphine (0.16 g; 0.61 mmol) were added. The mixture was stirred at room temperature for 48–52 h and monitored with TLC [72], as published by Lopes et al. [44]. Spectroscopic data for the compounds in this study are available in Supplementary Materials.

### 4.4. Cell Culture

As cells utilized were *L. braziliensis* amastigotes, *Plasmodium falciparum*, and U-937 (ATCC CRL- 1593.2TM) macrophages.

### 4.5. In Vitro Cytotoxicity

The compounds were analyzed for viability of the human pro-monocytic cell line U-937 (ATCC CRL- 1593.2TM) using an MTT (3-(4,5-dimethylthiazol-2-yl)-2,5- diphenyltetrazolium bromide) assay based on a previously described methodology, assessing cytotoxic activity [73]. The resulting compounds were determined by measuring mitochondrial dehydrogenase activity, adding 10 mL/well of MTT solution (0.5 mg/mL) and incubation at 37 °C for 3 h. Cell viability was defined from the quantity of formazan produced and the intensity of color (absorbance) registered through optical density (O.D.) obtained at 570 nm in a (VarioskanTM Flash Multimode Reader—Thermo Scientific, Waltham, MA, USA) spectrophotometer. As a negative control, isolated cells were cultured (without the compounds) to observe viability. Doxorubicin was used as a cytotoxicity control in a similar manner. We performed two independent tests with each concentration, in triplicate [73].

### 4.6. In Vitro Antileishmanial Activity

The activities of the derivatives in *L. braziliensis* intracellular amastigotes, transfected with the green fluorescent protein gene (MHOM/CO/88/UA301-EGFP) were evaluated. Initially, human U-937 cells at a density of 3 × 10^5^ cells/mL in RPMI 1640 and 0.1 μg/mL PMA (phorbol-12-myristate-13-acetate) were dispensed in a 24-well microplate and incubated at 37 °C, and 5% CO_2_. At 72 h of incubation, the cells were infected with growing (stationary phase) promastigotes; 15 parasites per cell. The plates were incubated at 34 °C and 5% CO_2_ for 3 h and the cells then washed twice with phosphate buffer solution (PBS) to eliminate non-internalized parasites. Fresh RPMI-1640 was added to each well (1 mL) and the plates were incubated again to complete the infection. At 24 h of infection, the RPMI-1640 medium was replaced by a fresh culture medium containing each compound in four serial dilutions, the highest concentration being equivalent to twice the LC_50_, and the plates were incubated at 37 °C and 5% CO_2_ for 72 h. The cells were then removed from the bottom plate with a trypsin/EDTA solution (250 mg), and centrifuged at 1100 rpm for 10 min at 4 °C; the supernatant was discarded and the cells were washed with 1 mL of cold PBS and centrifuged twice at 1100 rpm for 10 min at 4 °C. After this final wash, the supernatant was discarded and the cells were suspended in 500 μL of PBS.

The cells were run in a flow cytometer (Cytomics FC 500MPL), reading at 488 nm (exciting) and 525 nm (emitting) over an argon laser, and counting 10,000 events. Infected cells were determined according to green fluorescence events (parasites). Infected cells exposed to the control drug (amphotericin B) were used as antileishmanial activity controls (positive control). Infected cells incubated in the absence of any compound or drug were used as infection controls (negative control). Non-specific fluorescence was corrected by subtracting fluorescence from unstained cells. All determinations were performed in triplicate, in at least two independent experiments [74].

### 4.7. In Vitro Antiplasmodial Activity

Cultures of asynchronous *P. falciparum* 3D7 strains were adjusted for 0.5% parasitemia and 1% hematocrit in RPMI medium enriched with 3% lipid-rich bovine serum albumin—Albumax II. Afterwards, 100 µL of the parasite suspension was dispensed to each well of a 96-well cell culture plate and subsequently exposed to 100 µL of each compound at 100, 25, 6.25, or 1.56 µg/mL. The plates were incubated for 48 h at 37 °C in an atmosphere of N_2_ (90%), CO_2_ (5%) and O_2_ (5%). After incubation, the parasites were harvested and subjected to three 20-min freeze-thaw cycles. At the same time, 100 µL of Malstat reagent (400 µL of Triton X-100 in 80 mL of deionized water, 4 g of L-lactate, 1.32 g of Tris buffer, and 0.022 g of adenine acetylpyridine dinucleotide in 200 mL of deionized water; pH 9.0), and 25 mL of NBT/PES solution (0.16 g of nitroblue-tetrazolium salt, and 0.08 g of phenazine sulfate in 100 mL of deionized water) were added to each well of an additional 96-well plate. After freezing and thawing cycles, the culture in each of the wells on the first plate was re-suspended by pipetting, and 15 µL was removed from each well and added to the corresponding well on the additional plate (containing the Malstat and NBT/PES reagents). After one hour of incubation in the dark, the color development of the lactate dehidrogenase (LDH) reaction was read on a spectrofluorometer (Varioskan, Thermo, Waltham, MA, USA) at 650 nm. The color intensity in each experimental condition was recorded in fluorescent units (F.U). Non-specific fluorescence was corrected by subtracting F.U. from the blanks. The entire experiment was performed in triplicate in at least two independent experiments. Chloroquine (QC) was used as a positive control and the culture medium was used as a negative control [75].

### 4.8. Statistical Analysis

Cytotoxicity was determined according to the viability and mortality percentages obtained in each experimental condition (synthesized compounds, amphotericin B, benznidazole, chloroquine, and culture medium). Viability percentages were first calculated using Equation (1).

(1) $$\%\;Viability=\left(O.D\;exposed\;cells÷O.D\;Control\;cells\right)\;\times \;100$$

Then, cell growth inhibition percentage was calculated as 100 - % viability.

The cell growth inhibition percentages were used to calculate median lethal concentrations (LC_50_) using Probit analysis [76]. Toxicity was defined according to LC_50_ values, according to the following scale: toxic; LC_50_ < 100 μM; moderately toxic; LC_50_ > 100 μg/mL and <200 μM, and potentially nontoxic; LC_50_ > 200 μM. 

The antiplasmodial activity of each compound was evaluated through reduction in parasite growth, calculated according to Equation (2):

(2) $$\%\;parasite\;inhibition=100-\left[\left(F.U\;exposed\;parasites÷F.U\;non\;exposed\;parasites\right)\;\times \;100\right]$$

Data for percentages of parasite reduction or inhibition were used to calculate the EC_50_ using Probit linear regression modeling [76]. Activity was graded as high, moderate or low according to EC_50_ values as follows: high activity when the EC_50_ was <25 µg/mL; moderate activity when the EC_50_ was in the range from 25 to 50 µg/mL, and low activity when the EC_50_ was >50 μg/mL. The selectivity index (SI) was calculated dividing cytotoxic activity by anti-parasitic activity using the following formula: SI = CL_50_ ÷ CE_50_. 

### 4.9. Computational Methods

#### 4.9.1. Target Selection

Compound **9** (the most active) was selected for modeling studies. The potential target set for this compound was selected using a homology-based approach. Initially, potential targets of compound **9** were predicted using the similarity ensemble approach (SEA) web server [77], and the identified subset of potential targets was used as input for a BLAST search [78], for *Leishmania braziliensis* (taxid:5660) proteins found in the Reference Proteins Database (refseq_protein). The BLAST search was performed using its NCBI server implementation (https://blast.ncbi.nlm.nih.gov/). The *Leishmania braziliensis* proteins were covered (at a sequence minimum of 70%) by the BLAST sequence alignment. Those with at least 35% identity with any query protein were regarded as potential compound **9** parasite targets.

#### 4.9.2. Molecular Docking

Molecular docking followed the same protocol described in our previous publications [44,65]. The initial three-dimensional conformation of compound **9** was obtained with OpenEye Omega [79]; and AM1-BCC partial atomic charges were added to it using Molcharge [80]. Since none of the predicted targets had their 3D structures solved, homology models were generated for all the studied proteins with the SWISS-MODEL server [81]. Catalytically important cofactors, including metal ions, were added to the homology models taking their templates as reference.

Gold software was used for molecular docking [82]. Receptors’ binding sites were defined from the ligands present on the templates used for homology models. Any residue located at a distance lower than 6 Å from the reference ligand was selected for the active site. In the absence of a reference ligand, the binding site was defined as any amino acid within 10 Å of a point located at the center of the cavity. For primary docking, 30 different solutions were explored for compound **9** using the CHEMPLP scoring function with the Search efficiency of Gold set to 200%. The obtained 30 different ligand conformations were rescored with the GoldScore, ChemScore, and ASP scoring functions. Furthermore, during molecular docking the side chains of the residues pointing toward the binding cavity were set to flexible.

The most probable binding modes of compound **9** (for each receptor) were selected from a consensus scoring strategy. For this, the score of each ligand conformation according to scoring function j was converted to Z-scores following the transformation:

(3) $$Z_{i,j}=\frac{S_{i,j}-\overset{´}{S_{j}}}{std\left(S_{j}\right)}$$

where $Z_{i,j}$ is the Z-score of conformation *i* according to scoring function *j*, $\overset{´}{S_{j}}$ indicates the average value of scoring function *j* across all predicted binding modes and $std\left(S_{j}\right)$ represents the standard deviation of these values. The final aggregated score $Z_{i}$ of a predicted conformation was computed as the average of its Z-scores across all the CHEMPLP, GoldScore, ChemScore and ASP scoring functions. Any predicted binding mode with $Z_{i}>1$ was selected for further calculations.

#### 4.9.3. Molecular Dynamics Simulations and MM-PBSA Calculations

Amber 18 [83] was employed for molecular dynamics (MD) simulations and MM-PBSA calculations. These calculations were performed using the same protocol as in our previous publication [44]. For the MD simulations, all predicted ligand–receptor complexes underwent the same modeling steps: energy minimization, heating, equilibration, production runs, and free energy of binding prediction. The Amber force field ff14SB and gaff force fields were respectively used to parameterize amino acids and non-amino acid residues. MD simulations took place in an explicit solvent for which the complexes were enclosed in truncated octahedron boxes and solvated with TIP3P water molecules. To neutralize any excess net charge, either Na^+^ of Cl^−^ ions were added to the solvated systems.

The prepared systems were energy minimized in two steps. First, 500 steps of the steepest descent method followed by 500 cycles of conjugate gradient were executed while constraining all atoms except solvent with a force constant of 500 kcal/mol·Å^2^. Then, all atoms were unconstrained for the 500 steps of the steepest descent algorithm followed by 1000 cycles of conjugate gradient. Long range electrostatic interactions were treated using the particle mesh Ewald (PME) method implemented in Amber, and a cutoff distance of 10 Å during all minimization steps.

The systems were then heated from 0 K to 300 K, keeping their volumes constant at 20 ps. The solute was constrained with a force constant of 10 kcal/mol·Å^2^ and temperature was controlled using a Langevin thermostat with a collision frequency of 1.0 ps^−1^ during heating. The PME cutoff was set to 12 Å during this modeling stage. During all MD steps from this point onwards, the bonds involving hydrogen atoms were constrained and their interactions omitted by means of the SHAKE algorithm.

The heated systems were then equilibrated for 100 ps, at constant pressure (1 bar) and temperature (300 K). For equilibration, temperature was controlled during heating, and pressure was controlled with isotropic position scaling set at a relaxation time of 2 ps. The PME cutoff for equilibration was set to 12 Å. The equilibrated systems were used as inputs for 20 production runs of 2 ns length each, using the same parameters as those used during the equilibration of the systems. Atomic velocities were randomly initialized before each production run. 

Compound **9** free energy of binding to its potential receptors was estimated using MM-PBSA calculations. These were performed with the MM-PBSA.py script provided with Amber 18 [84]. The MM-PBSA calculations were performed with 200 MD snapshots (one every 200 ps) selected from 20 production runs with the ionic strength set to 100 mM.

## Figures and Tables

**Table 1:** Antileishmanial and antiplasmodial activity, cytotoxicity and molar mass of compounds **1**–**12**.

*p*-Coumaric Acid Esters						
	*Leishmania braziliensis* Amastigotes		*Plasmodium falciparum*		U-937 Macrophages	Molar Mass (g/mol)
	EC_50_ (μg/mL)^a^	SI	EC_50_ (μg/mL)^b^	SI	LC_50_ (μg/mL)	
**1**	8.28 ± 0.14	0.76	64.59 ± 2.89	0.1	6.26 ± 0.03	178.18
**2**	4.91 ± 0.64	0.21	110.31 ± 30.84	0.01	1.06 ± 1.08	192.21
**3**	6.3 ± 1.14	1.89	139.04 ± 4.07	0.09	11.89 ± 0.8	206.23
**4**	28.33 ± 0.27	3.4	106.08 ± 9.97	0.91	96.23 ± 18.89	206.23
**5**	86.98 ± 18.13	1.46	98.66 ± 17.7	1.28	126.71 ± 16.5	222.23
**6**	323.29 ± 50.04	0.1	198.84 ± 7.9	0.16	30.9 ± 6.32	220.26
**7**	32.03 ± 5.05	0.5	95.48 ± 1.84	0.17	16.09 ± 3.81	234.29
**8**	6.02 ± 0.22	3.25	156.37 ± 8.2	0.13	19.58 ± 1.76	234.29
**9**	4.14 ± 0.55	2.72	206.35 ± 46.4	0.05	11.24 ± 0.86	248.31
**10**	16.34 ± 0.49	0.94	81.19 ± 8.54	0.19	15.43 ± 1.84	332.47
**11**	5.69 ± 0.3	3.66	83.98 ± 15.34	0.25	20.81 ± 3.47	268.30
**12**	11.21 ± 0.2	3.14	93.66 ± 14.17	0.38	35.2 ± 13.94	296.36
AMB^c^	0.3 ± 0.07	128			38.4 ± 6.3	
CQ^d^			3.35 ± 0.39	14.1	47.26 ± 4.9	

Data represent mean value ± standard deviation. ^a^ LC_50_: median lethal concentration; ^b^ EC_50_: median effective concentration; ^c^ AMB: amphotericin B, antileishmanial drug control ^d^ CQ: chloroquine diphosphate salt, antimalarial drug control.

**Table 2:** Potential targets of compound **9** in *Leishmania braziliensis* identified by the homology-based target fishing approach.

*L. braziliensis* Target ^(a)^	Description	ID
A4HED7_LEIBR	Serine/threonine-protein kinase	PKC
A4HE56_LEIBR	Aldehyde dehydrogenase, mitochondrial	ALDH2
A4HP40_LEIBR	Aldehyde dehydrogenase	ALDH
A4HG21_LEIBR	Aldo-keto reductase protein	AKR
A4HGT2_LEIBR	DNA topoisomerase 2	TOP2
A4H9D1_LEIBR	Putative glycogen synthase kinase	GSK3
A4HBR4_LEIBR	Histone deacetylase	HDA1
A4HDK5_LEIBR	Histone deacetylase	HDA2
A4HA94_LEIBR	Mitogen-activated protein kinase	MPK4
A4H9X2_LEIBR	Mitogen-activated protein kinase	CBPKC
A4HJJ7_LEIBR	Prostaglandin-F synthase protein	PGFS
A4HK26_LEIBR	Prostaglandin f synthase	PGFS2
A4H9L8_LEIBR	Protein kinase A catalytic subunit	PKAC3
A4HN71_LEIBR	Protein kinase A catalytic subunit isoform 1	PKAC2a
A4HEL2_LEIBR	Protein kinase domain-containing protein	CRK7
A4H8N9_LEIBR	Tyrosyl or methionyl-tRNA synthetase-like protein	YARS1

^(a)^ UniProt entry name.

**Table 3:** Results of the molecular docking of compound **9** to its predicted potential molecular targets in *Leishmania braziliensis*.

Target	Conformer	CHEMPLP		GoldScore		ChemScore		ASP		Consensus Z-Score
		Score	Z-Score	Score	Z-Score	Score	Z-Score	Score	Z-Score	
PKC	1	64.76	2.69	29.03	1.06	24.23	1.32	24.27	1.68	1.69
	2	60.60	1.31	29.05	1.07	23.04	0.71	23.60	1.35	1.11
ALDH2	1	63.77	2.22	34.27	1.46	21.22	0.76	25.99	0.47	1.23
ALDH	1	64.64	1.97	66.89	1.54	20.48	1.65	27.38	0.79	1.49
	2	59.38	0.56	69.68	2.24	20.25	1.54	27.18	0.71	1.26
	3	62.51	1.40	66.04	1.32	17.22	0.09	29.32	1.63	1.11
	4	62.25	1.33	65.00	1.06	17.98	0.45	29.19	1.57	1.10
AKR	1	60.32	2.04	10.04	1.10	25.09	2.50	37.69	2.35	2.00
	2	61.32	2.17	−8.41	0.13	21.15	1.84	26.77	1.04	1.30
	3	51.69	0.90	−2.36	0.45	18.34	1.37	29.66	1.39	1.03
TOP2	1	57.19	1.26	33.54	1.29	19.27	1.92	23.73	1.36	1.46
	2	57.11	1.24	32.47	1.01	16.63	0.85	22.31	0.92	1.00
GSK3 ^(a)^	1	70.94	2.37	34.80	1.27	24.32	1.97	32.04	2.70	2.08
	2	67.43	1.62	29.45	0.22	23.34	1.40	29.85	1.66	1.22
GSK3 SBS ^(a)^	1	50.52	1.64	25.70	0.50	13.30	0.87	18.51	1.87	1.22
	2	48.54	1.17	27.61	0.72	14.49	1.32	17.12	1.34	1.14
HDA1	1	65.54	1.28	29.53	0.57	24.30	0.79	31.58	1.40	1.01
HDA2	1	67.10	0.94	40.20	1.55	27.36	1.03	35.93	1.33	1.21
MPK4	1	58.40	2.11	32.17	1.73	20.59	0.66	20.43	0.70	1.30
	2	55.89	1.23	26.35	0.12	24.14	1.92	21.72	1.27	1.14
	3	57.14	1.67	33.77	2.17	20.50	0.63	18.31	−0.25	1.05
CBPKC	1	54.89	2.04	24.42	1.07	17.09	0.06	19.03	0.89	1.01
PGFS	1	50.11	1.89	24.68	1.70	13.91	1.14	30.81	1.82	1.64
PGFS2	1	32.76	1.06	13.87	1.33	7.74	1.27	20.71	1.51	1.29
PKAC3	1	58.29	1.47	36.02	1.16	23.65	1.83	24.29	1.08	1.39
	2	57.86	1.33	31.27	0.57	21.71	1.01	26.05	1.82	1.18
	3	56.43	0.84	36.64	1.24	21.06	0.73	24.93	1.35	1.04
PKAC2a	1	61.50	2.15	32.33	1.09	24.27	1.72	20.13	−0.17	1.20
CRK7	1	66.15	2.13	36.34	2.04	24.01	1.60	25.98	1.43	1.80
	2	65.15	1.86	34.07	1.38	23.39	1.36	24.01	0.65	1.31
	3	63.92	1.53	33.69	1.27	21.55	0.62	25.55	1.26	1.17
YARS1	1	61.62	2.08	21.92	1.15	21.95	1.56	19.98	0.23	1.26
	2	57.26	0.97	16.22	0.80	21.32	1.24	23.33	1.52	1.13

^(a)^ Two different binding sites were explored for GSK3: the ATP binding site (GSK3) and the substrate binding pocket (GSK3 SBS).

**Table 4:** Predicted free energies of binding of compound **9** to its predicted targets. The components of the free energies of binding are also included. All energies are expressed in kcal/mol.

Target	Conformer	MM-PBSA Components							ΔG TOTAL^(h)^
		VDWAALS^(a)^	EEL^(b)^	EPB^(c)^	ENPOLAR^(d)^	EDISPER^(e)^	ΔG Gas^(f)^	ΔG Solv^(g)^	
PKC	1	−31.35	−27.22	37.23	−27.27	43.17	−58.57	53.12	−5.45
	2	−33.93	−15.64	29.63	−28.73	46.01	−49.57	46.91	−2.66
ALDH2	1	−29.85	−26.61	36.34	−25.56	43.08	−56.46	53.86	−2.60
ALDH	1	−38.10	−15.73	36.01	−29.50	48.51	−53.83	55.02	1.19
	2	−37.59	−22.81	36.17	−30.23	49.25	−60.40	55.19	−5.21
	3	−34.69	−23.13	37.63	−28.16	46.95	−57.82	56.43	−1.39
	4	−38.78	−30.30	40.58	−30.43	50.68	−69.08	60.83	−8.25
AKR	1	−30.88	−13.99	28.85	−25.79	40.67	−44.87	43.74	−1.13
	2	−28.48	−7.63	22.63	−22.28	36.82	−36.11	37.17	1.06
	3	−29.47	−13.77	32.42	−23.82	39.14	−43.24	47.74	4.50
TOP2	1	−42.66	−24.71	42.55	−31.82	51.87	−67.37	62.60	−4.76
	2	−46.13	−16.83	35.84	−31.37	52.34	−62.96	56.80	−6.15
GSK3	1	−28.68	−11.34	31.25	−21.69	37.74	−40.02	47.30	7.27
	2	−34.81	−17.30	30.70	−28.87	46.12	−52.11	47.95	−4.16
GSK3 (SBS)	1	−18.60	−14.21	23.44	−16.13	26.89	−32.81	34.20	1.39
	2	−27.34	−9.22	26.18	−20.94	35.99	−36.56	41.23	4.67
HDA1	1	−28.68	−16.20	31.32	−22.81	37.85	−44.88	46.36	1.47
HDA2	1	−25.51	−24.37	35.37	−22.39	35.83	−49.88	48.80	−1.08
MPK4	1	−30.88	−24.72	35.63	−26.12	42.49	−55.60	51.99	−3.61
	2	−36.54	−13.69	31.88	−29.36	46.60	−50.23	49.11	−1.11
	3	−39.58	−17.44	32.41	−30.01	47.48	−57.02	49.88	−7.14
CBPKC	1	−32.78	−22.89	35.50	−27.42	44.34	−55.67	52.42	−3.25
PGFS	1	−26.09	−9.33	19.97	−20.90	34.35	−35.42	33.41	−2.01
PGFS2	1	−22.24	−11.63	20.66	−18.94	30.60	−33.87	32.32	−1.55
PKAC3	1	−36.72	−34.98	57.32	−29.95	47.33	−71.70	74.69	2.99
	2	−38.00	−12.24	33.67	−28.84	47.80	−50.25	52.63	2.38
	3	−37.74	−34.00	57.82	−30.26	47.61	−71.74	75.18	3.44
PKAC2a	1	−33.06	−7.86	27.04	−25.35	43.07	−40.92	44.76	3.84
CRK7	1	−40.13	−18.73	38.07	−31.07	48.14	−58.86	55.14	−3.72
	2	−34.61	−12.95	30.38	−27.47	45.17	−47.56	48.08	0.52
	3	−38.51	−15.27	35.40	−30.09	48.31	−53.78	53.62	−0.16
YARS1	1	−25.99	−11.84	23.28	−21.71	35.63	−37.83	37.19	−0.64
	2	−24.81	−17.62	28.89	−22.35	34.35	−42.43	40.89	−1.54

^(a)^ van der Waals energy, ^(b)^ electrostatic energy, ^(c)^ polar solvation energy, ^(d)^ non-polar solvation energy, ^(e)^ dispersion solvation energy, ^(f)^ total gas phase free energy, ^(g)^ total solvation free energy, ^(h)^ total free energy.

## Supplementary Materials

Supplementary materials can be found at https://www.mdpi.com/1420-3049/25/14/3178/s1.
