# Supplementary material for: Alkyl and Aryl Derivatives Based on p-Coumaric Acid Modification and Inhibitory Action against Leishmania braziliensis and Plasmodium falciparum
Source: Molecules. 2020 Jul 11;25(14):3178. doi: 10.3390/molecules25143178 (PMC7397144; doi:10.3390/molecules25143178)
Supplement: Supplementary file 1 [file molecules-25-03178-s001.pdf]

Supplementary materials

Alkyl and Aryl Derivatives Based on *p*-Coumaric Acid Modification and Inhibitory Action against *Leishmania braziliensis* and *Plasmodium falciparum*

Susiany P. Lopes [1], Lina M. Yepes [2], Yunierkis Pérez-Castillo [3], Sara M. Robledo [2] and Damião P. de Sousa [1,4,*]

[1] PostGraduation Program in Technological Development and Innovation in Medicines, Federal University of Paraíba, João Pessoa CEP 58051-970, Brazil; susiany.lopes@academico.ufpb.br
[2] PECET-Facultad de Medicina, Universidad de Antioquia, Medellín Calle 70 # 52-21, Colombia; lina.yepes@udea.edu.co (L.M.Y.); sara.robledo@udea.edu.co (S.M.R.)
[3] Escuela de Ciencias Físicas y Matemáticas, Universidad de Las Américas, Quito 170504, Ecuador; yunierkis.perez@udla.edu.ec
[4] Department of Pharmaceutical Sciences, Federal University of Paraíba, João Pessoa CEP 58051-970, Brazil
* Correspondence: damiao_desousa@yahoo.com.br

Received: 22 May 2020; Accepted: 17 June 2020; Published: date

Abstract: In low-income populations, neglected diseases are the principal cause of mortality. Of these, Leishmaniasis and malaria, being parasitic, protozoan infections, affect millions of people worldwide and are creating a public health problem. The present work evaluates the leishmanicidal and antiplasmodial action of a series of twelve *p*-coumaric acid derivatives. Of the tested derivatives, eight presented antiparasitic activities **1-3**, **8-12**. The hexyl *p*-coumarate derivative (**9**) (4.14 $\pm$ 0.55 μg/mL; Selectivity Index (SI) = 2.72) presented the highest leishmanicidal potency against the *Leishmania braziliensis* amastigote form. The results of the molecular docking study suggest that this compound inhibits aldehyde dehydrogenase (ALDH), mitogen activated kinase protein (MPK4), and DNA topoisomerase 2 (TOP2), all of which are key enzymes in the development of *Leishmania braziliensis*. The data indicate that these enzymes interact via van der Waals bonds, hydrophobic interactions, and hydrogen bonds with phenolic and aliphatic parts of this same compound. Of the other compounds analyzed, methyl *p*-coumarate (64.59 $\pm$ 2.89 μg/mL; IS = 0.1) presented bioactivity against *Plasmodium falciparum*. The study reveals that esters presenting a *p*-coumarate substructure are promising for use in synthesis of derivatives with good antiparasitic profiles.

Keywords: hydroxycinnamic acids; natural products; leishmanicidal activity; antiplasmodial activity; cytotoxicity; neglected diseases

1. Experimental

1.1. Chemical Characterization Compounds 1-12.

(*E*)-Methyl 3-(4-hydroxyphenyl)acrylate (**1**): white solid; Yield 87.98% (95.5 mg, 0.53 mmol); m.p.: 135-136 °C [1]; TLC (7:3 hexane/EtOAc), R_f = 0.52; IR υmax (KBr, cm^{-1}): 3379, 3045, 2954, 1737, 1634, 1601, 1434, 1284, 1170; ^{1}H NMR (DMSO-d_6, 400 MHz): δH 3.69 (3H; *s*), 6.38 (1H; *d*; *J* =16 Hz), 6.80-6.78 (1H; *d*; *J*=8.60 Hz), 7.55-7.52 (1H; *d*; *J*=8.44 Hz), 7.58 (1H; *s*); ^{13}C NMR (DMSO-d_6, 100 MHz): δC 51.3, 114.0, 115.8, .125.2, 130.4, 144.9, 159.9, 167.1 [2,3].

(*E*)-Ethyl 3-(4-hydroxyphenyl)acrylate (**2**): white solid; Yield 84.21% (295.8 mg, 1.53 mmol); m.p.: 76-77 °C; TLC (7:3 hexane/EtOAc), R_f = 0.55; IR υmax (KBr, cm^{-1}): 3286, 3026, 2985, 1683, 1634, 1604, 1440, 1280, 1169; ^{1}H NMR ($CDCl_3$, 400 MHz): δH 1.33 (3H; *t*; *J*=7.16 Hz), 4.26 (2H; *quart*, *J*=7.16Hz),

6.28 (1H; *d*; *J*=15.94 Hz), 6.88-6.86 (2H; *d*; *J*=8.64 Hz); 7.40-7.38 (2H, *d*, *J*=8.64 Hz); 7.63 (1H; *d*; *J*=15.96 Hz); ^{13}C NMR ($CDCl_3$, 100 MHz): δC 14.4, 61, 115.0, 116.1, 126.8, 130.2, 145.5, 158.7, 168.5 [1, 4, 5].

(*E*)-Propyl 3-(4-hydroxyphenyl)acrylate (**3**): White solid; Yield 58.43% (220 mg, 1.06 mmol); m.p.: 74-75 °C; TLC (7:3 hexane/EtOAc), R_f = 0.57; IR υmax (KBr, cm^{-1}): 3376, 2970, 2879, 1674, 1638, 1603, 1441, 1271, 1171; ^{1}H NMR ($CDCl_3$, 400 MHz): δH 0.99 (3H; *t*; *J*=7.4 Hz), 1.73 (2H; *sext*; *J*=7.44), 4.16 (2H; *t*; *J*=6.72 Hz), 6.30 (1H; *d*; *J*=15.95 Hz); 6.87-6.85 (2H, *d*, *J*=8.66 Hz); 7.43- 7.40 (2H; *d*; *J*=8.67 Hz), 7.63(1H, *d*, *J*=15.96 Hz); ^{13}C NMR ($CDCl_3$, 100 MHz): δC 10.8, 22.2, 66.5, 115.4, 116.0, 127.1, 130.22, 145.0, 158.3, 168.5 [1,2].

(*E*)-Isopropyl 3-(4-hydroxyphenyl)acrylate (**4**): White solid; Yield 60.73% (228.9 mg, 1.10 mmnol); m.p.: 71-72 °C; TLC (7:3 hexane/EtOAc), R_f = 0.57; IR υmax (KBr, cm^{-1}): 3272, 2980, 2939, 1675, 1629, 1605, 1466, 1279, 1172; ^{1}H NMR ($CDCl_3$, 400 MHz): δH 1.31 (6H; *d*; *J*=6.28 Hz), 5.14 (1H; *hept*; *J*=6.25Hz), 6.27 (1H; *d*; *J*=15.94 Hz); 6.89-6.87 (2H, *d*, *J*=8.60 Hz); 7.40-7.38 (2H; *d*; *J*=8.67 Hz), 7.61(1H, *d*, *J*=15.95 Hz); ^{13}C NMR ($CDCl_3$, 100 MHz): δC 22.2, 68.4, 115.6, 116.1, 126.8, 130.3, 145.0, 158.7, 167.1 [1,2].

(*E*)-2-methoxyethyl 3-(4-hydroxyphenyl)acrylate (**5**): white solid; Yield 42.76% (173.7 mg, 0.78 mmol) ; m.p.: 96-97 °C; TLC (7:3 hexane/EtOAc), R_f = 0.33; IR υmax (KBr, cm^{-1}): 3260, 3043, 2959, 1696, 1655, 1609, 1447, 1266, 1176; ^{1}H NMR ($CDCl_3$, 400 MHz): δH 3.46 (3H; *s*), 3.71 (2H; *t*, *J*=4.64 Hz), 4.37 (2H, *t*, *J*=4.52 Hz), 6.21 (1H, *d*, *J* =15.94 Hz), 6.83-6.81 (2H, *d*; *J*=8.55 Hz), 7.35- 7.32 (2H, *d*, *J*=8.7 Hz), 7.60 (1H, *d*, *J* =15.95 Hz) ^{13}C NMR ($CDCl_3$, 100 MHz): δC 58.8, 63.2, 114.6, 116.1, 126.5, 130.1, 145.6, 158.8, 167.8 [1,2].

(*E*)-Butyl 3-(4-hydroxyphenyl)acrylate (**6**): Brown amorphous; Yield 98% (395.5 mg, 1.79 mmol) ; TLC (7:3 hexane/EtOAc), R_f = 0.65; IR υmax (KBr, cm^{-1}): 3277, 2959, 2873, 1683, 1634, 1604, 1474, 1280, 1172; ^{1}H NMR ($CDCl_3$, 400 MHz): δH 0.96 (3H; *t*; *J*=7.38 Hz), 1.44 (2H; *sext*; *J*=7.44), 1.69 (2H; *pent*; *J*=6.80 Hz), 4.20 (3H; *t*, *J*=6.67); 6.30 (1H; *d*; *J*=15.94 Hz); 6.86-6.84 (2H, *d*, *J*=8.59 Hz); 7.43-7.41 (2H; *d*; *J*=8.59 Hz), 7.62 (1H, *d*, *J*=15.95 Hz); ^{13}C NMR ($CDCl_3$, 100 MHz): δC 14.0, 19.3, 30.8, 64.8, 115.2, 116.1, 126.8, 130.2, 145.2, 158.5, 168.5 [1,2].

(*E*)-Pentyl 3-(4-hydroxyphenyl)acrylate (**7**): Brown amorphous; Yield 44.09% (188.8 mg, 0.80 mmol) ; TLC (7:3 hexane/EtOAc), R_f = 0.60; IR υmax (KBr, cm^{-1}): 3400, 2959, 2872, 1685, 1633, 1605, 1445, 1268, 1170; 1H NMR ($CDCl_3$, 400 MHz): δH 0.94 (3H, *t*, *J*=6.99), 1.40 (2H, *m*), 1.40 (2H, *m*), 1.73 (2H, *quint*, *J*=7.32 Hz), 4.22 (2H, *t*, *J*=6.74 Hz), 6.32 (1H, *d*, *J*=15.95 Hz), 6.90-6.88 (2H, *d*; *J*=8.62 Hz), 7.44-7.42 (2H, *d*, *J*=8.62 Hz), 7.65 (1H, *d*, *J*=15.94 Hz) ^{13}C NMR ($CDCl_3$, 100 MHz): δC 14.1, 22.5, 28.2, 28.5, 65.1, 115.2, 116.1, 126.9, 130.2, 145.1, 158.5, 168.4 [1,2].

(*E*)-Isopentyl 3-(4-hydroxyphenyl)acrylate (**8**): Brown solid; Yield 79.36% (339.8 mg, 1.45 mmol); m.p.: 111-112 °C (lit. 151-153 °C, [1]); TLC (7:3 hexane/EtOAc), R_f = 0.66; IR υmax (KBr, cm^{-1}): 3380, 2958, 2873, 1687, 1636, 1604, 1437, 1277, 1167; ^{1}H NMR ($CDCl_3$, 400 MHz): δH 0.95 (3H, *d*, *J*=6.64 Hz), 1.72 (1H, *quart*, *J*=6,75 Hz), 1.60 (2H, *d*, *J*= 6,74 Hz), 4.23 (2H, *t*, *J*=6.83 Hz), 6.31 (1H, *d*, *J*=15.96 Hz,), 6.88-6.86 (2H, *d*; *J*=8.44 Hz), 7.41-7.39 (2H, *d*, *J*=8.49 Hz), 7.64 (1H, *d*, *J*=15.98 Hz) ^{13}C NMR ($CDCl_3$, 100 MHz): δC 22.6, 25.3, 37.5, 63.6, 115.2, 116.1, 126.8, 130.2, 145.1, 158.6, 168.6 [1,2].

(*E*)-Hexyl 3-(4-hydroxyphenyl)acrylate (**9**): White solid; Yield 36.88% (167.4 mg; 0.67 mmol); m.p.: 42-43 °C (lit. 160 °C, [7]); TLC (8:2 hexane/EtOAc), R_f = 0.47; IR υmax (KBr, cm^{-1}): 3383, 2955, 2857, 1675, 1625, 1604, 1472, 1276, 1170; ^{1}H NMR ($CDCl_3$, 400 MHz): δH 0.89 (3H, *t*, *J*=6.02), 1.36 (6H, *m*), 1.69 (2H, *quint*; *J*=16.0 Hz), 4.19 (2H, *t*, *J*=6.74 Hz), 6.28 (1H, *d*, *J*=15.94 Hz,), 6.88-6.86 (2H, *d*; *J*=8.64 Hz), 7.42-7.40 (2H, *d*, *J*=8.62 Hz), 7.63 (1H, *d*, *J*=15.95 Hz) ^{13}C NMR ($CDCl_3$, 100 MHz): δC 14.1, 22.7, 25.8, 28.8, 31.6, 65.1, 115.4, 116.1, 127, 130.1, 145.0, 158.4, 168.3 [1,5, 7].

(*E*)-Dodecyl 3-(4-hydroxyphenyl)acrylate (**10**): White solid; Yield 29.55% (179.6 mg, 0.54 mmol); m.p.: 76-77 °C (lit. 73-75 °C, [6]); TLC (8:2 hexane/EtOAc), R_f = 0.52; IR υmax (KBr, cm^{-1}): 3381, 2926, 2848, 1674, 1625, 1603, 1473, 1275, 1171; ^{1}H NMR ($CDCl_3$, 400 MHz): δH 0.88 (3H, *t*, *J*=6.68),1.35 (18H, *m*), 1.70 (2H, *quint*, *J* = 6.72 Hz), 4.20 (2H, *t*, *J* = 6.76 Hz), 6.30 (1H, *d*, *J*=15.96 Hz,), 6.88-6.86 (2H, *d*; *J*=8.64 Hz), 7.42-7.40 (2H, *d*, *J*=8.64 Hz), 7.63 (1H, *d*, *J*=15.96 Hz) ^{13}C NMR ($CDCl_3$, 100 MHz): δC 14.1, 22.7, 26, 28.7, 29.3, 29.4, 29.5, 29.6, 29.6, 29.7, 31.9, 64.9, 115.3, 116, 126.9, 130, 145.1, 158.5, 168.5 [1,6].

(*E*)-4-Methylbenzyl 3-(4-hydroxyphenyl)acrylate (**11**): White solid; Yield 30% (147.2 mg, 0.54 mmol); m.p.: 93-94 °C; TLC (8:2 hexane/EtOAc), R_f = 0.34; IR υmax (KBr, cm^{-1}): 3291, 3027, 2958, 1689, 1629, 1605, 1450, 1279, 1164; ^{1}H NMR ($CDCl_3$, 400 MHz): δH 2.37(3H, *s*), 5.22 (2H, *s*), 6.33 (1H, *d*, *J*=

15.94 Hz), 6.86-6.84 (2H, *d*; *J*=8.76 Hz), 7.20-7.18 (2H, *d*, *J*=7.8 Hz), 7.32-7.30 (2H, *d*, *J*=8.04 Hz), 7.40-7.38 (2H, *d*, *J*=8.72 Hz), 7.66 (1H, *d*, *J*=15.96 Hz) ^{13}C NMR ($CDCl_3$, 100 MHz): δC 21.7, 66.6, 115.1, 116.1, 127.0, 128.6, 129.4, 130.2, 133.1,138.3, 145.5, 158.3, 168.0 [1,8].

(*E*)-4-Isopropylbenzyl 3-(4-hydroxyphenyl)acrylate (**12**): White solid; Yield 29.76% (161.2 mg, 0.54 mmol); m.p.: 107-108 °C; TLC (8:2 hexane/EtOAc), R_f = 0.39; IR υmax (KBr, cm^{-1}): 3232, 3031, 2960, 1709, 1638, 1605, 1455, 1226, 1158; ^{1}H NMR ($CDCl_3$, 400 MHz): δH 1,27 (6H, *d*,*J*=6.92 Hz), 2.94 (1H, *sept*, *J*= 6,74Hz), 5.24 (2H, *s*), 6.35 (1H, *d*, *J*=15.94 Hz), 6.87-6.85 (2H, *d*; *J*=8.55 Hz),7.28 (2H,*m*), 7.38-7.36 (2H,*d*, *J*=8.2), 7.43-7.41 (2H, *d*, *J*=8.4 Hz), 7.69 (1H, *d*, *J*=15.96 Hz) ^{13}C NMR ($CDCl_3$, 100 MHz): δC 24.1, 34.2, 66.6, 128.6, 126.8, 149.3, 126.8, 128.6, 133.5, 115.2, 116.1, 127.1, 130.2, 145.3, 158.2, 167.9 [1,8].

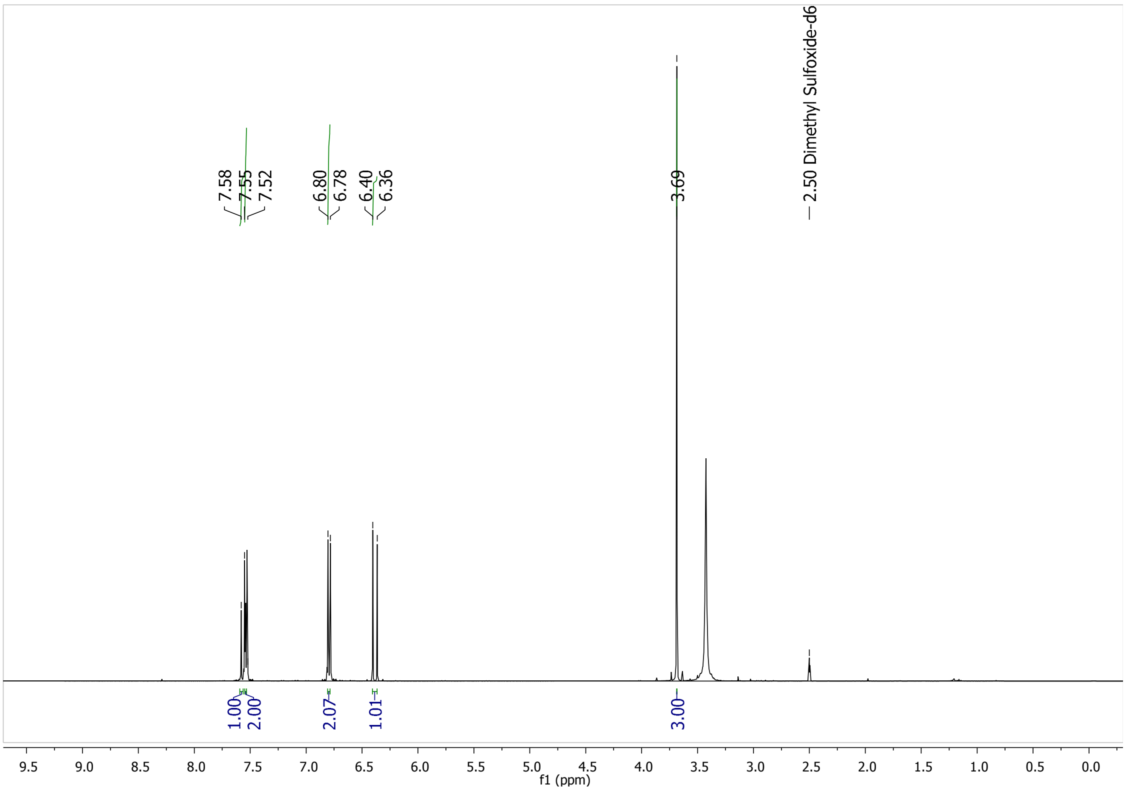

Figure S1. ^{1}H NMR (DMSO-d_6, 400 MHz) spectrum of methyl *p*-coumarate (**1**).

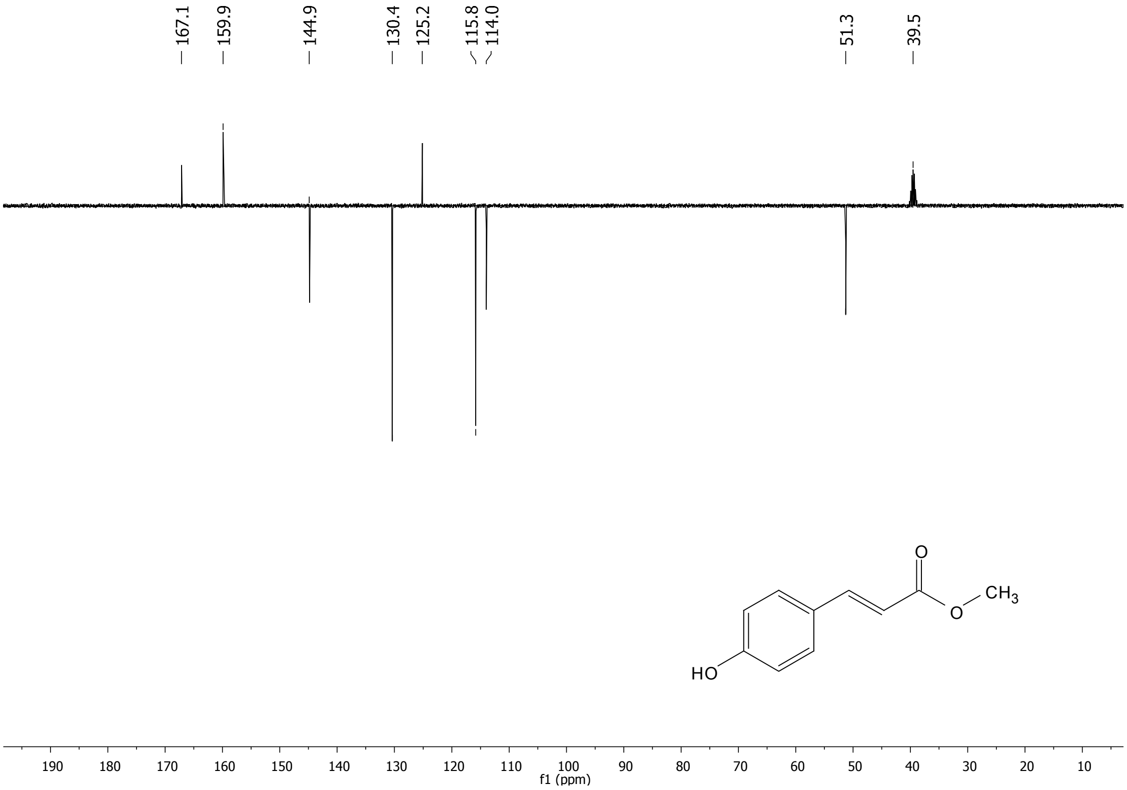

Figure S2. ^{13}C NMR (DMSO-d$_6$, 100 MHz) spectrum of methyl *p*-coumarate (**1**).

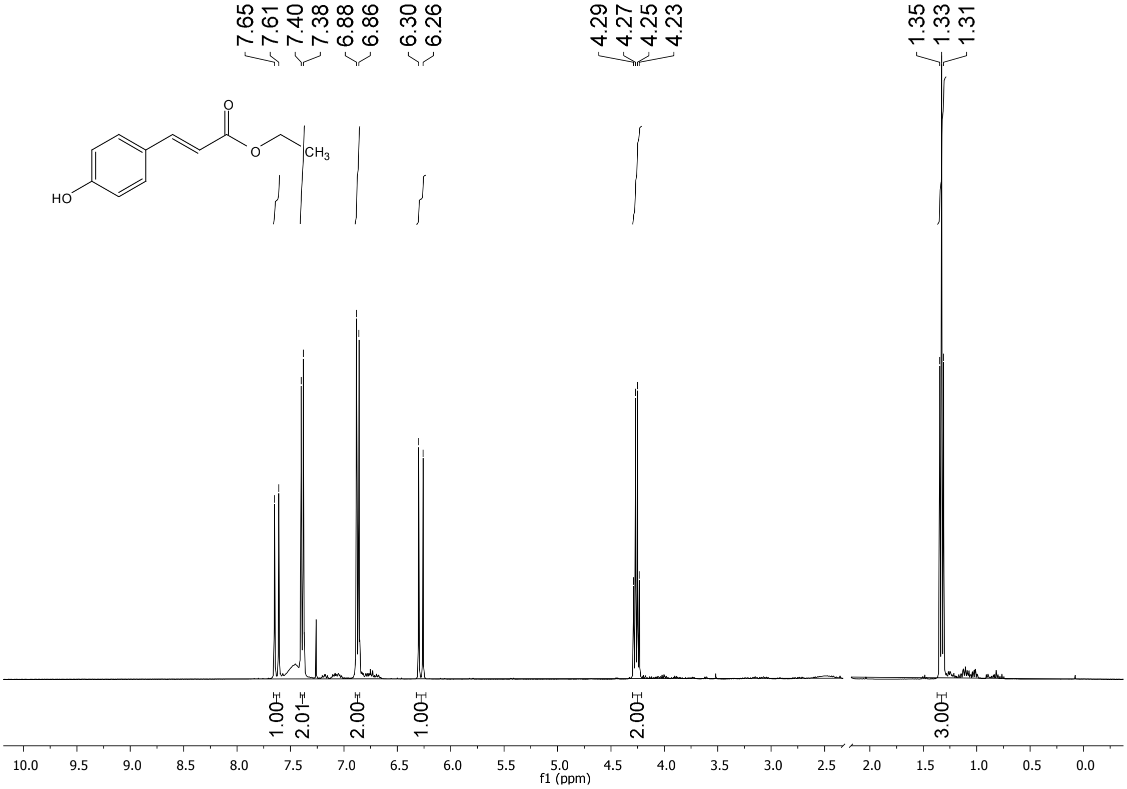

Figure S3. ^{1}H NMR (CdCl$_3$, 400 MHz) spectrum of ethyl *p*-coumarate (**2**).

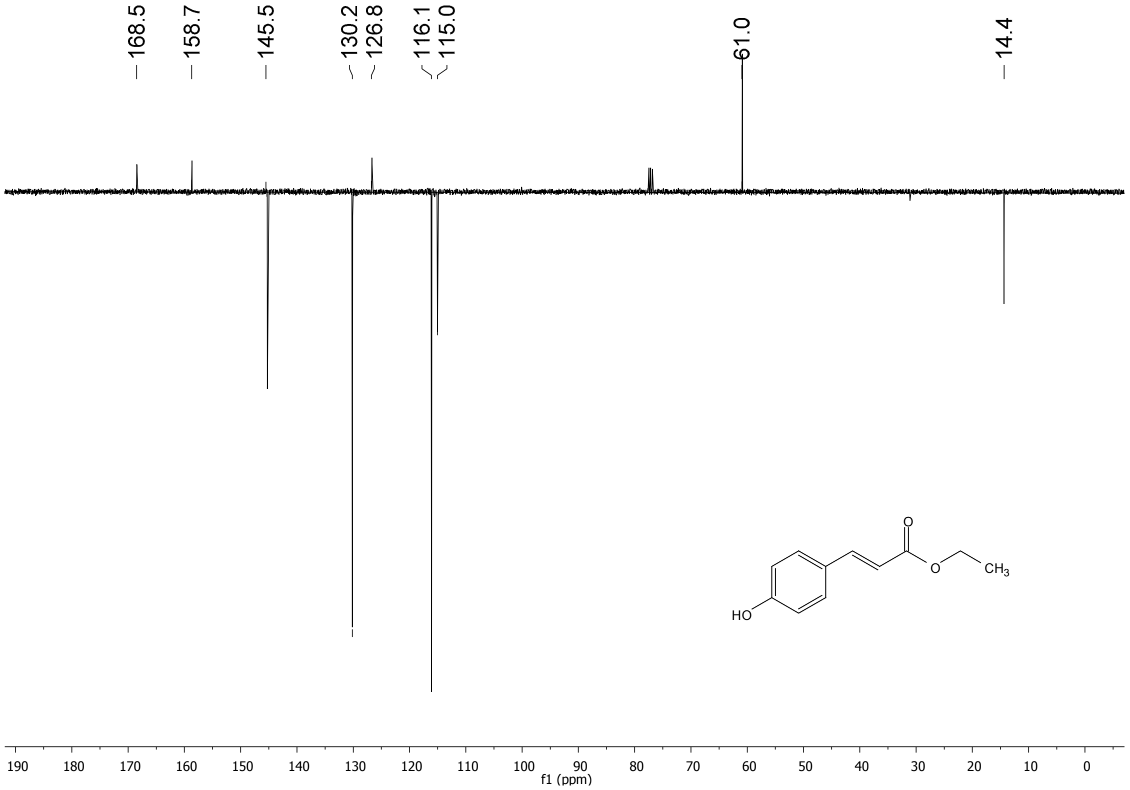

Figure S4. ^{13}C NMR ($CdCl_3$, 100 MHz) spectrum of ethyl *p*-coumarate (**2**).

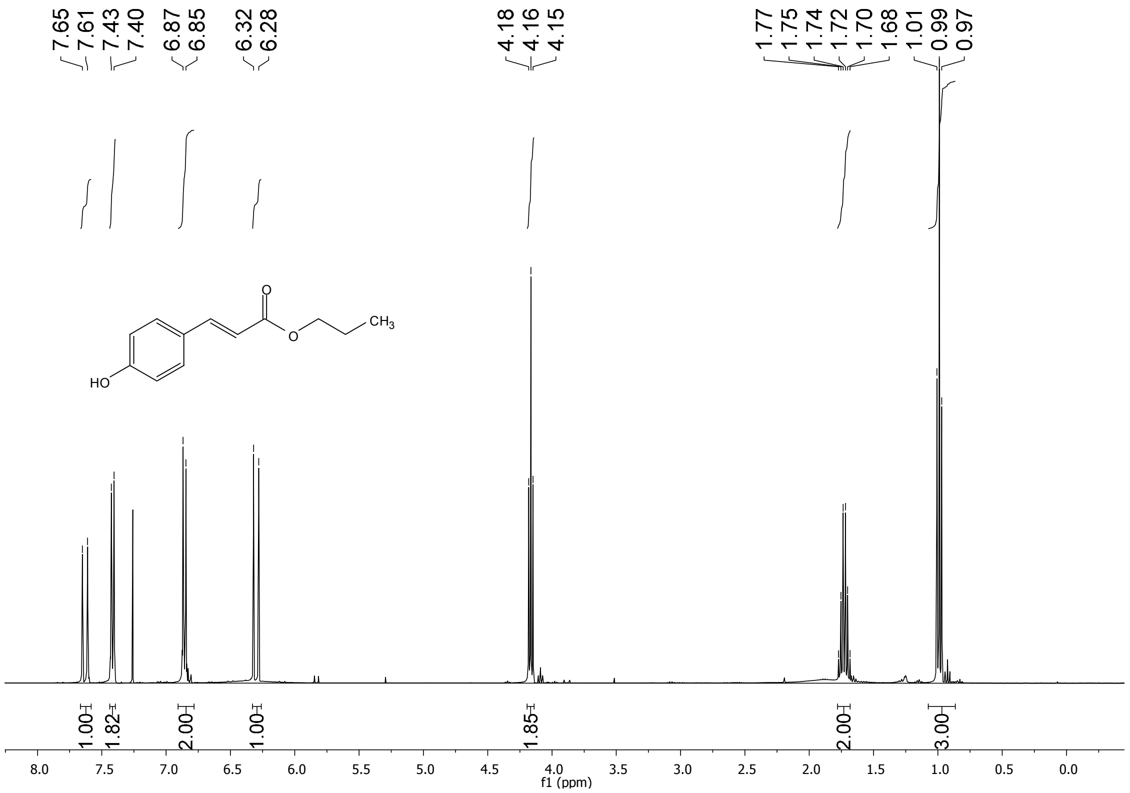

Figure S5. ^{1}H NMR ($CdCl_3$, 400 MHz) spectrum of propyl *p*-coumarate (**3**).

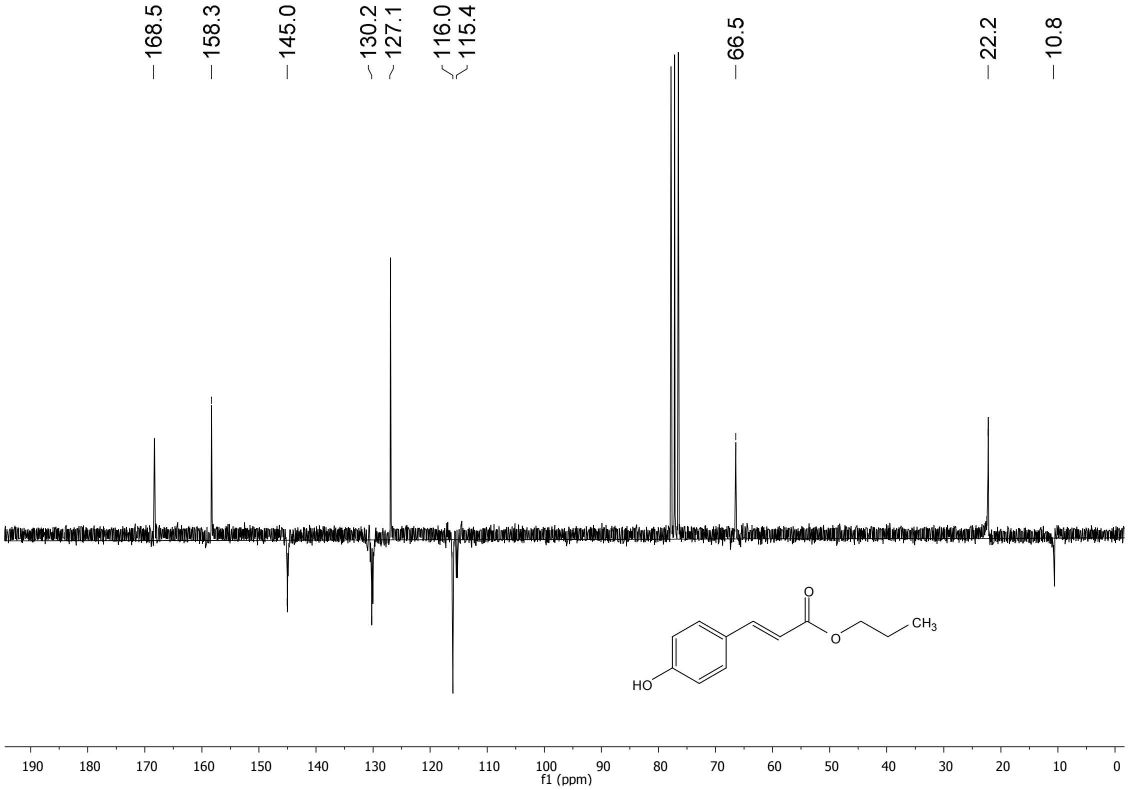

Figure S6. ^{13}C NMR (CdCl$_3$, 100 MHz) spectrum of propyl *p*-coumarate (**3**).

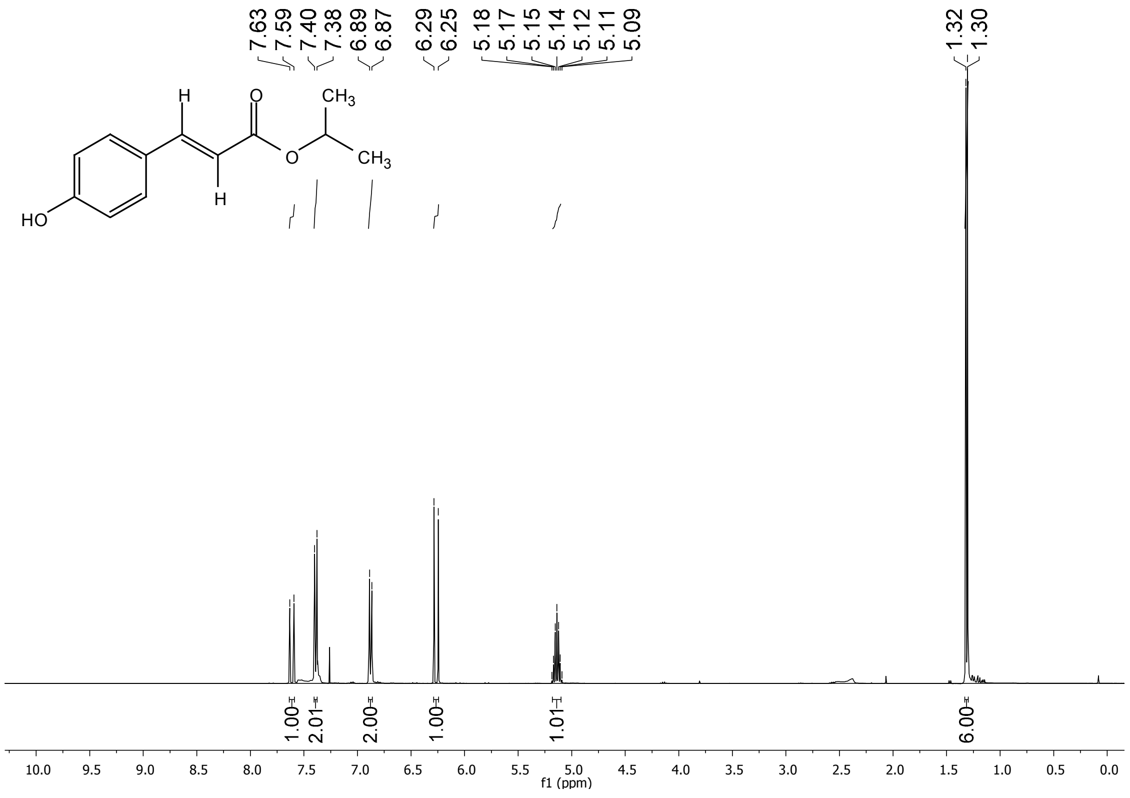

Figure S7. ^{1}H NMR (CdCl$_3$, 400 MHz) spectrum of isopropyl *p*-coumarate (**4**).

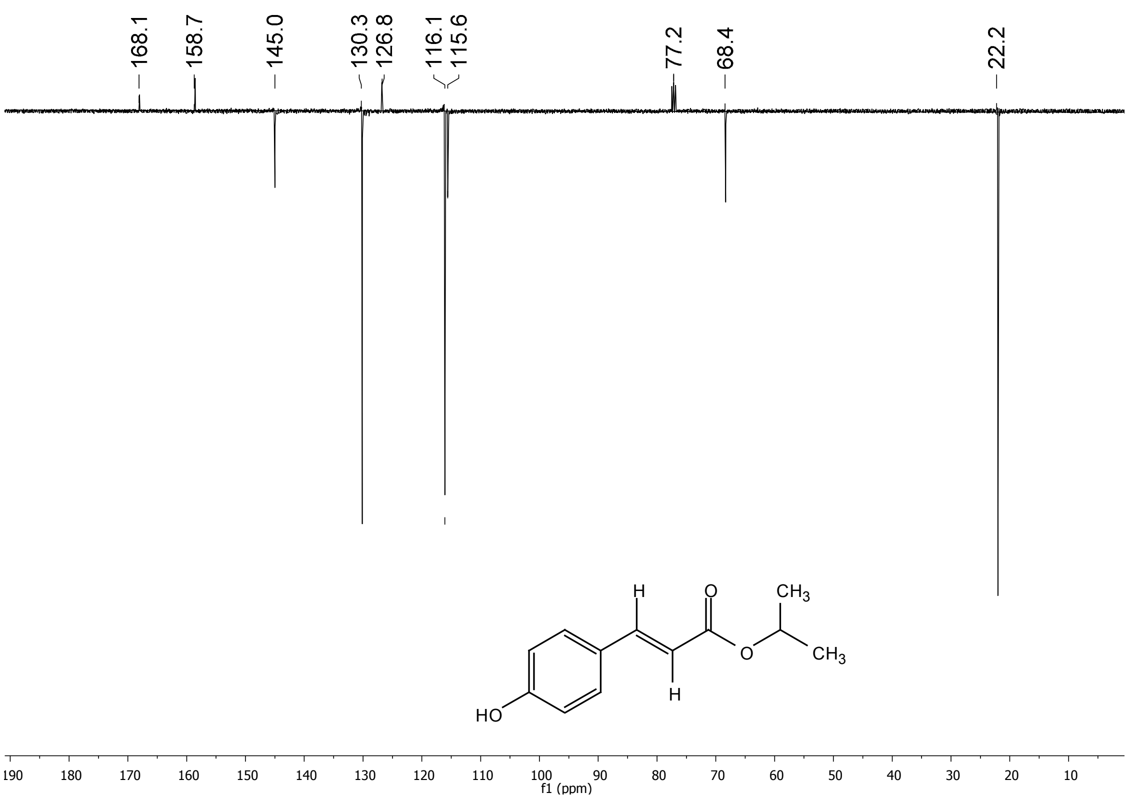

Figure S8. ^{13}C NMR (CdCl3, 100 MHz) spectrum of isopropyl *p*-coumarate (**4**).

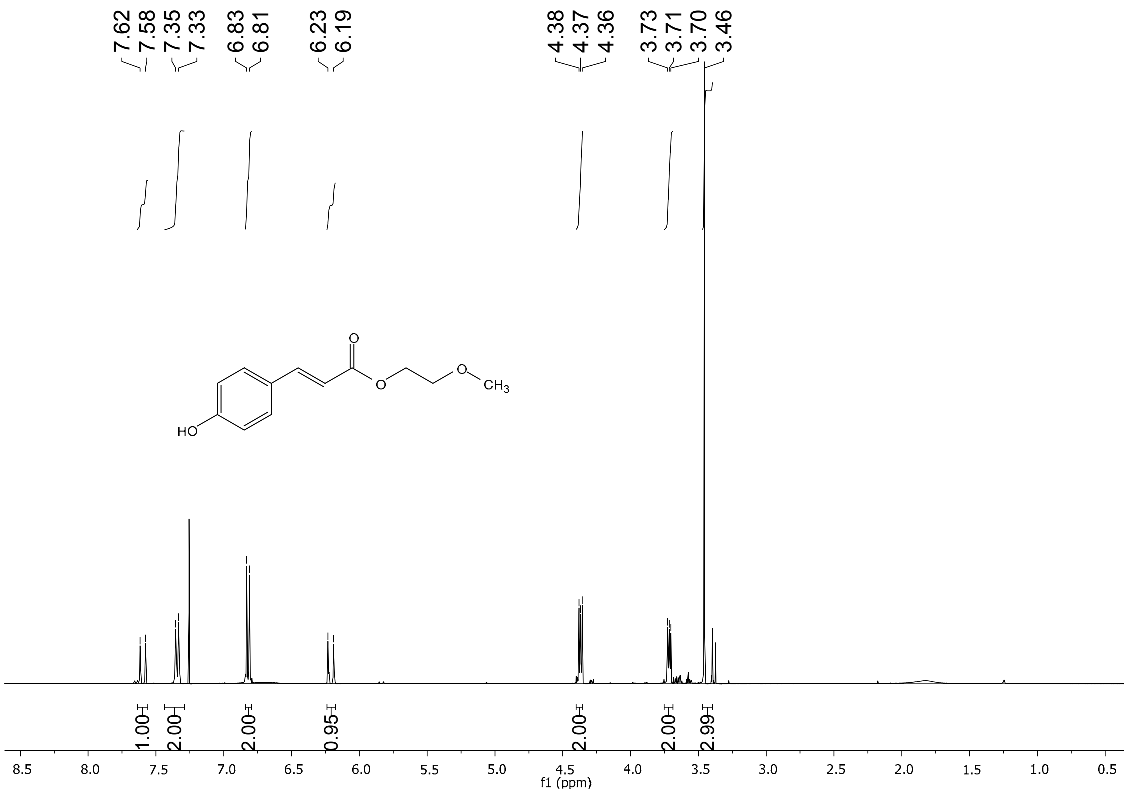

Figure S9. ^{1}H NMR (CdCl3, 400 MHz) spectrum of Methoxyethyl *p*-coumarate (**5**).

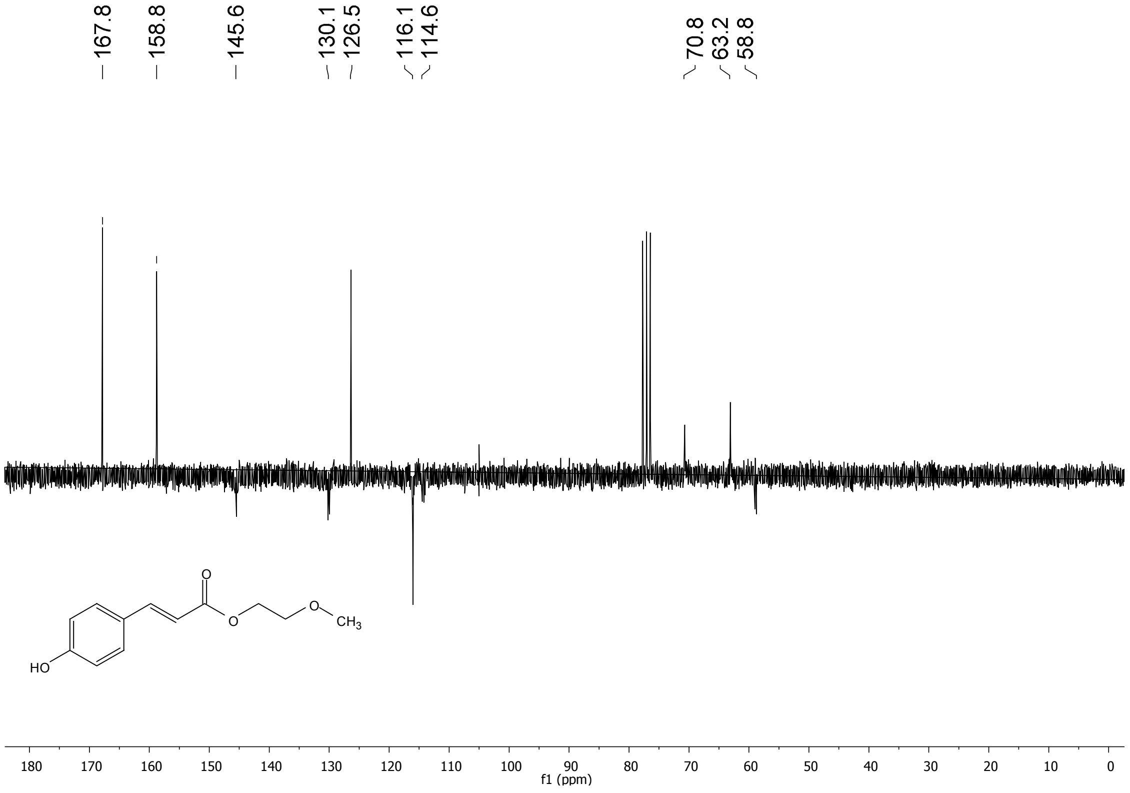

Figure S10. ^{13}C NMR ($CdCl_3$, 100 MHz) spectrum of Methoxyethyl *p*-coumarate (**5**).

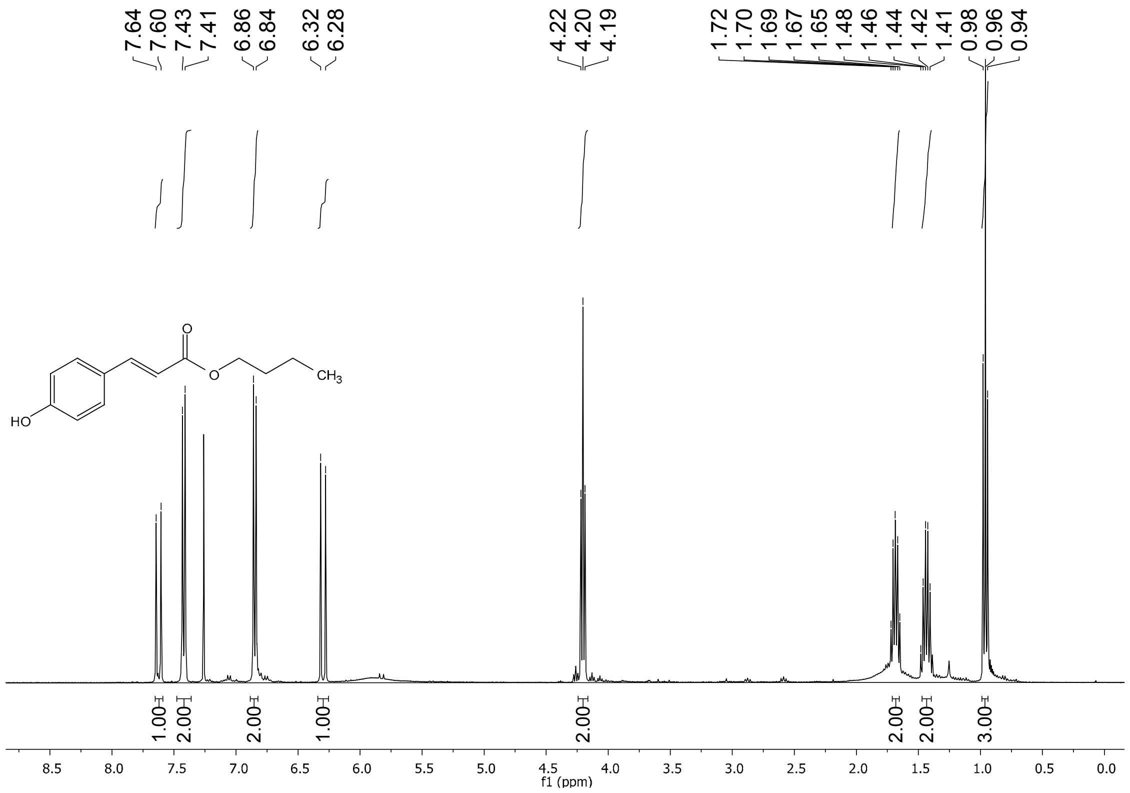

Figure S11. ^{1}H NMR ($CdCl_3$, 400 MHz) spectrum of butyl *p*-coumarate (**6**).

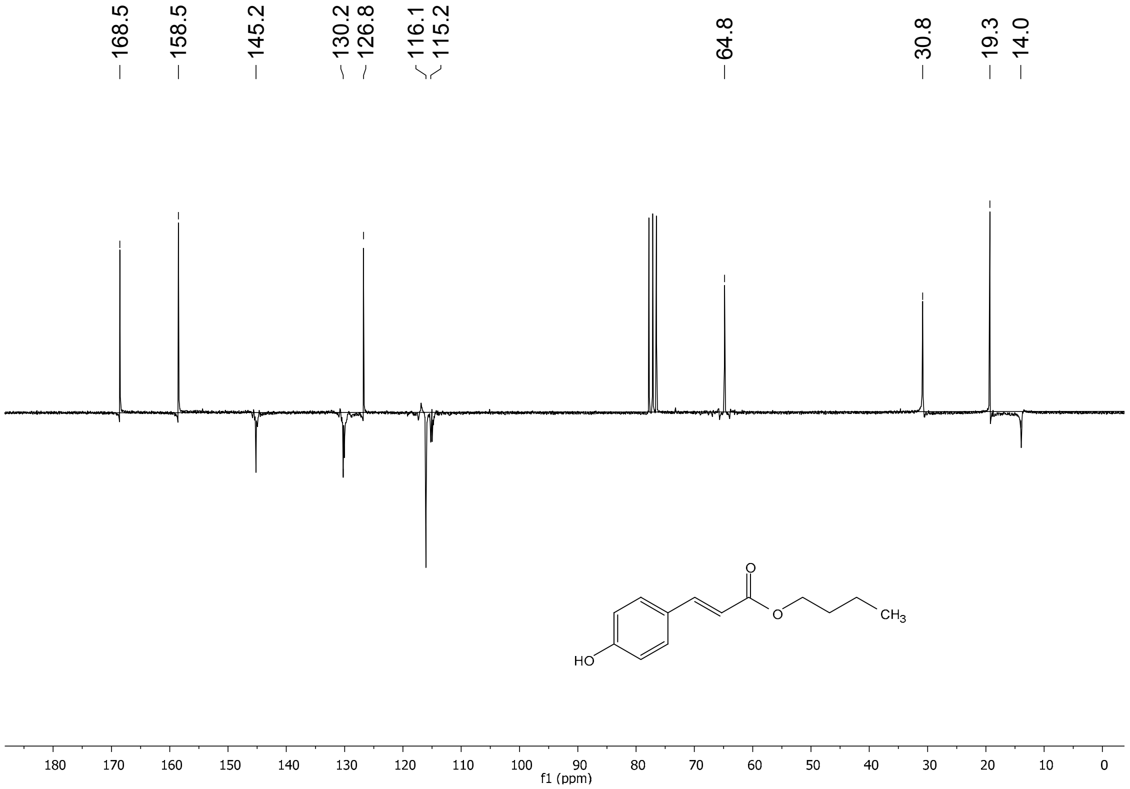

Figure S12. ^{13}C NMR (CdCl3, 100 MHz) spectrum of butyl *p*-coumarate (**6**).

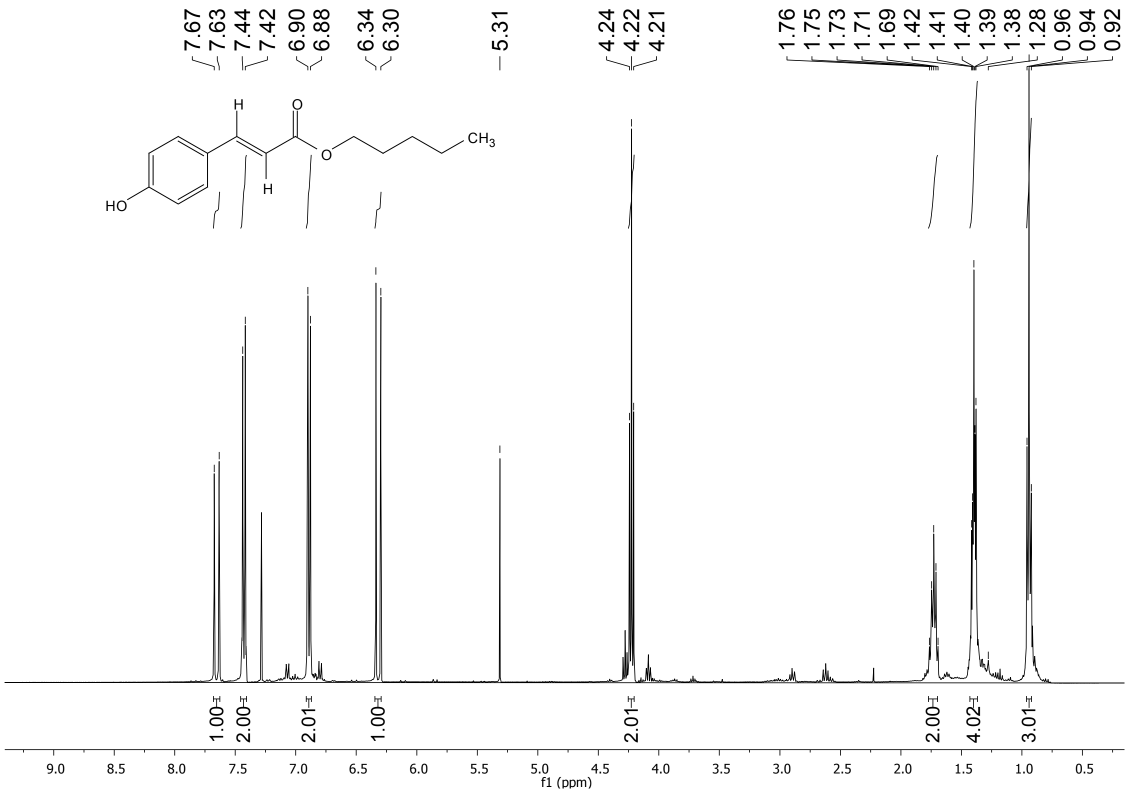

Figure S13. ^{1}H NMR (CdCl3, 400 MHz) spectrum of pentyl *p*-coumarate (**7**).

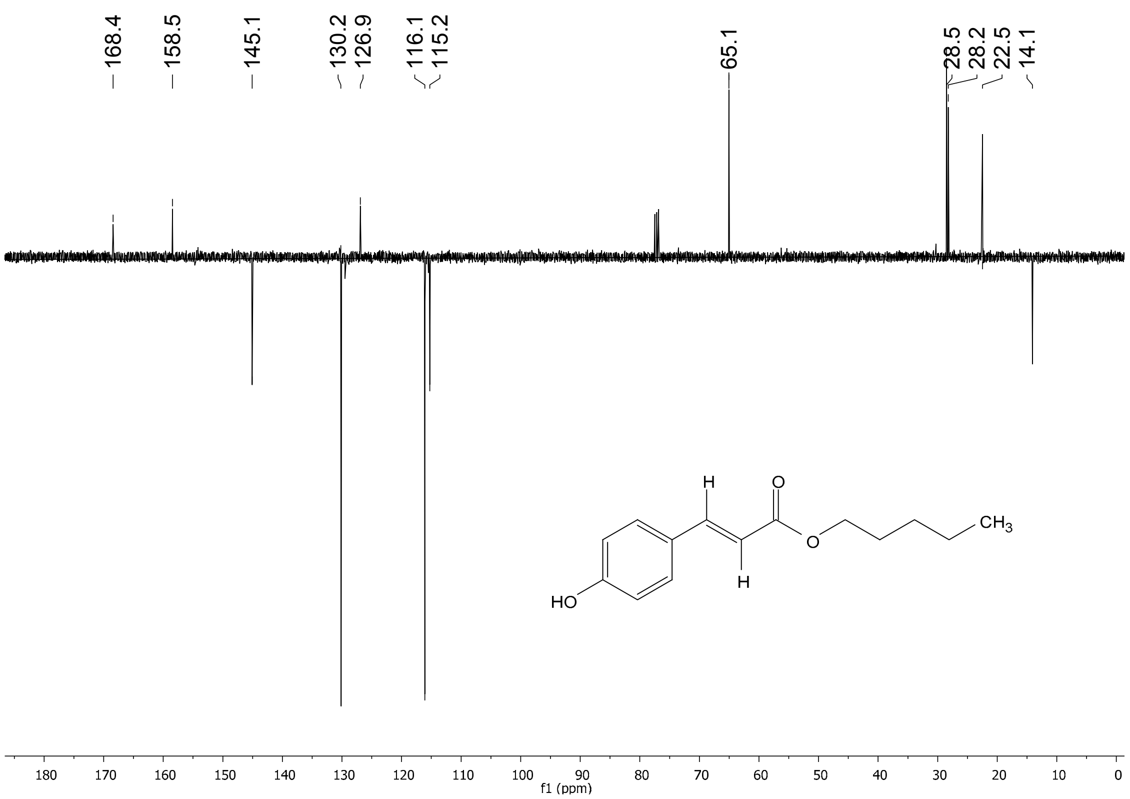

Figure S14. ^{13}C NMR (CdCl3, 100 MHz) spectrum of pentyl *p*-coumarate (**7**).

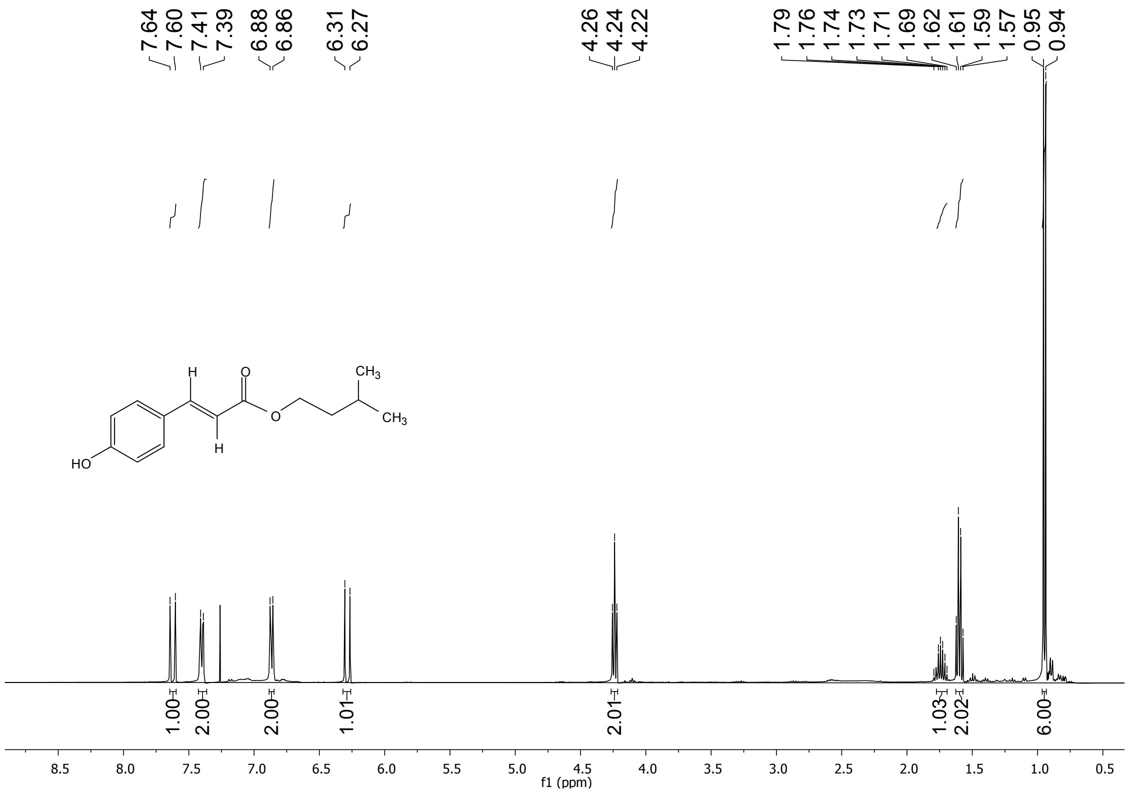

Figure S15. ^{1}H NMR (CdCl3, 400 MHz) spectrum of isopentyl *p*-coumarate (**8**).

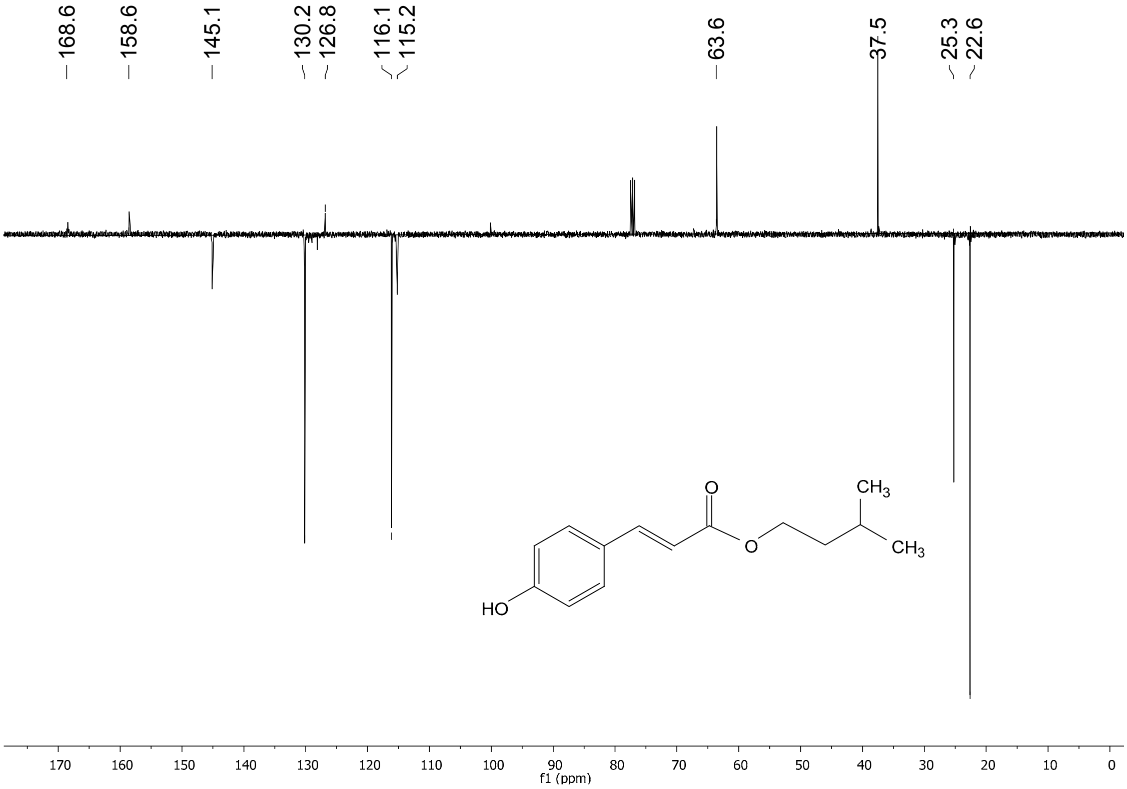

Figure S16. ^{13}C NMR ($CdCl_3$, 100 MHz) spectrum of isopentyl *p*-coumarate (**8**).

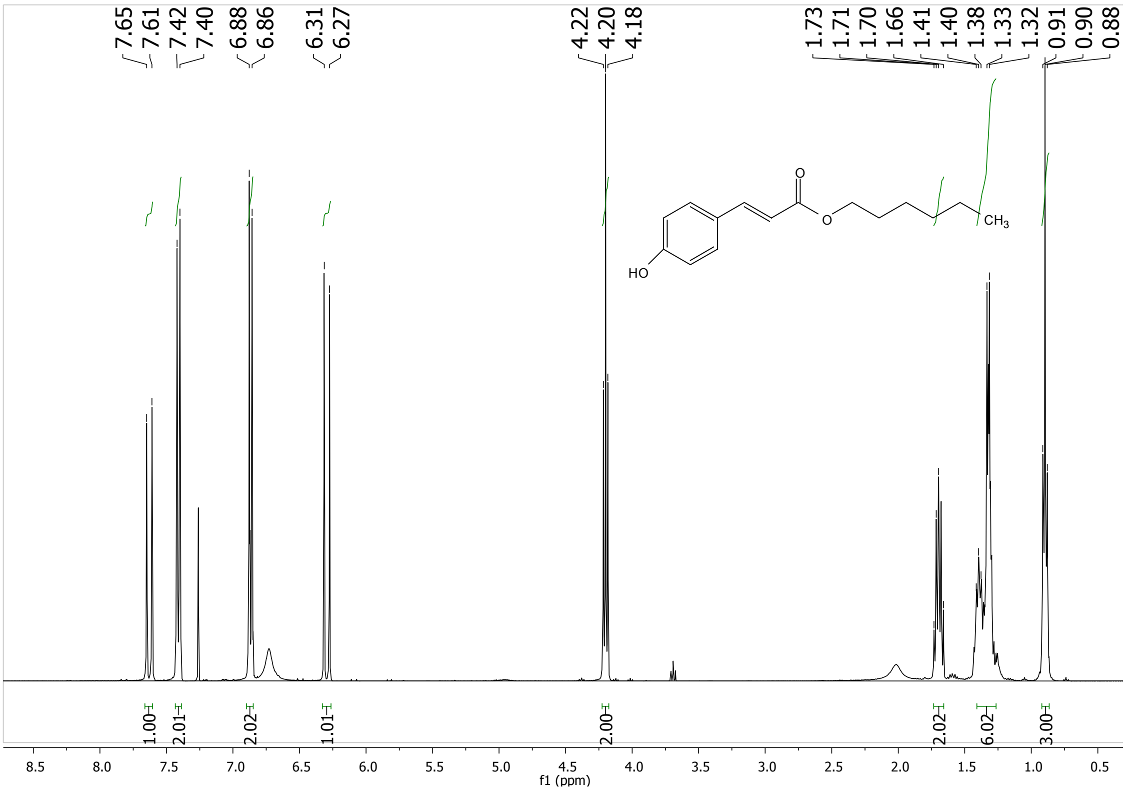

Figure S17. ^{1}H NMR ($CdCl_3$, 400 MHz) spectrum of hexyl *p*-coumarate (**9**).

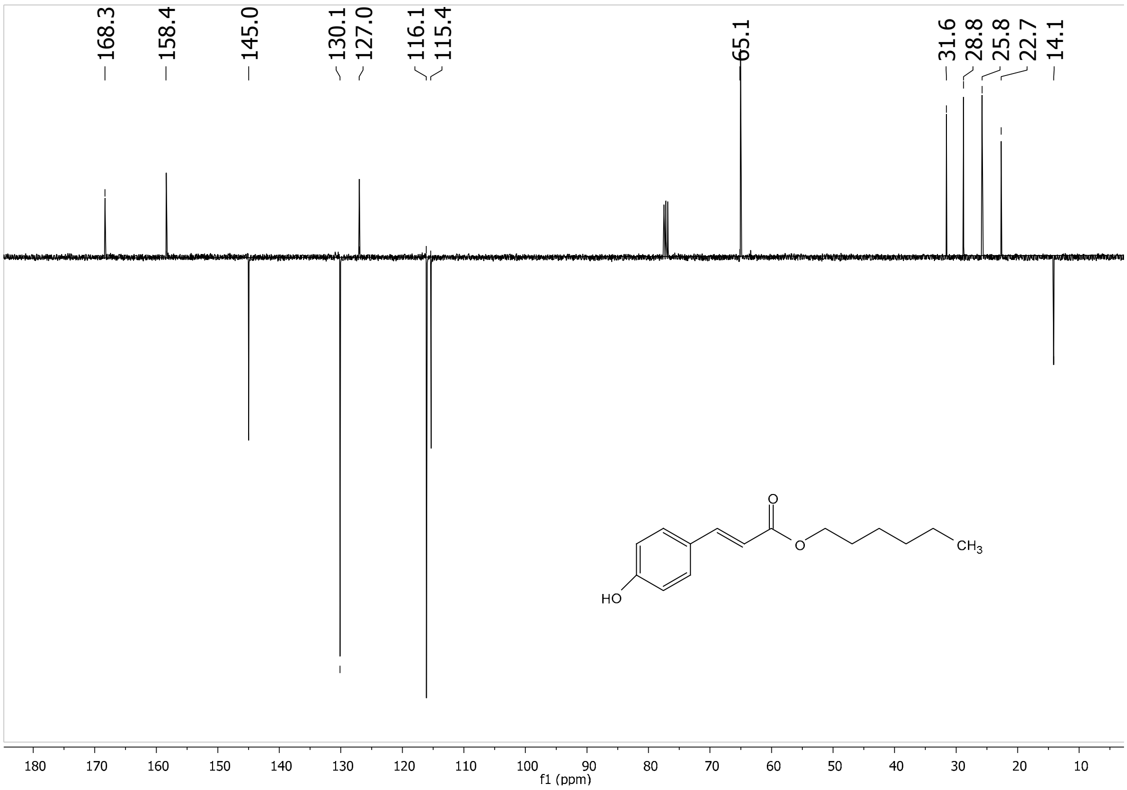

Figure S18. ^{13}C NMR ($CdCl_3$, 100 MHz) spectrum of hexyl *p*-coumarate (**9**).

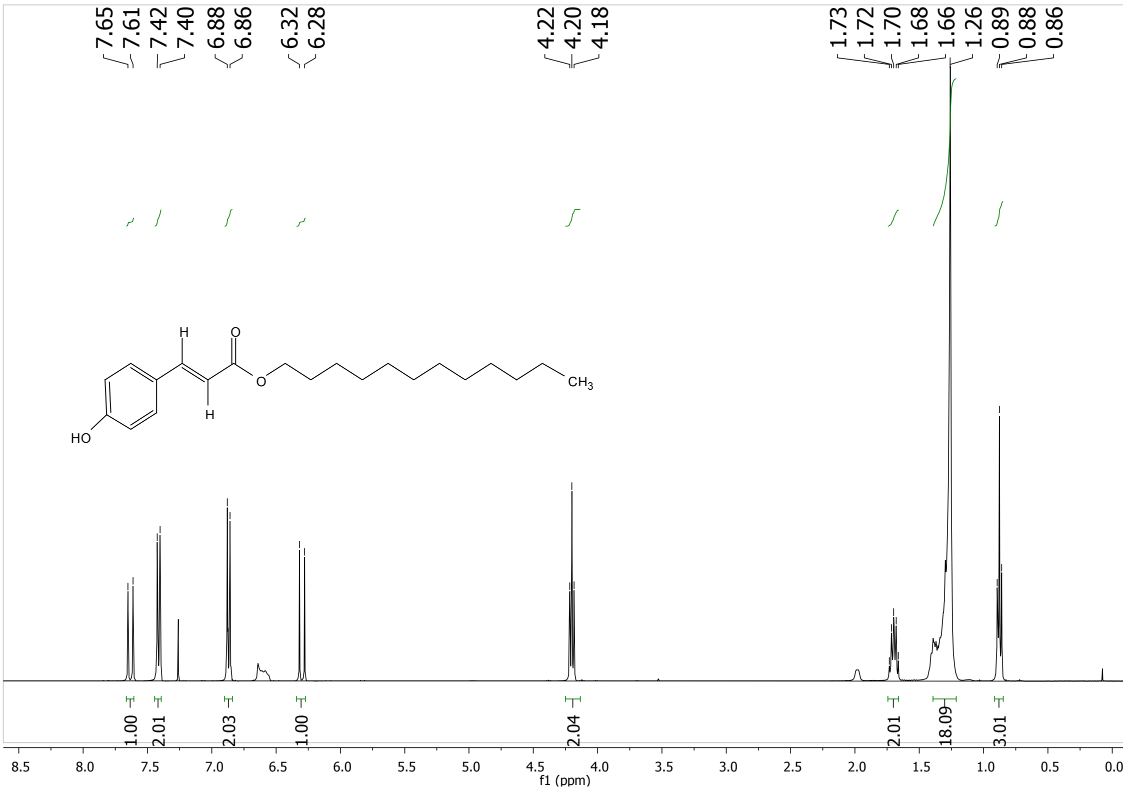

Figure S19. ^{1}H NMR ($CdCl_3$, 400 MHz) spectrum of dodecyl *p*-coumarate (**10**).

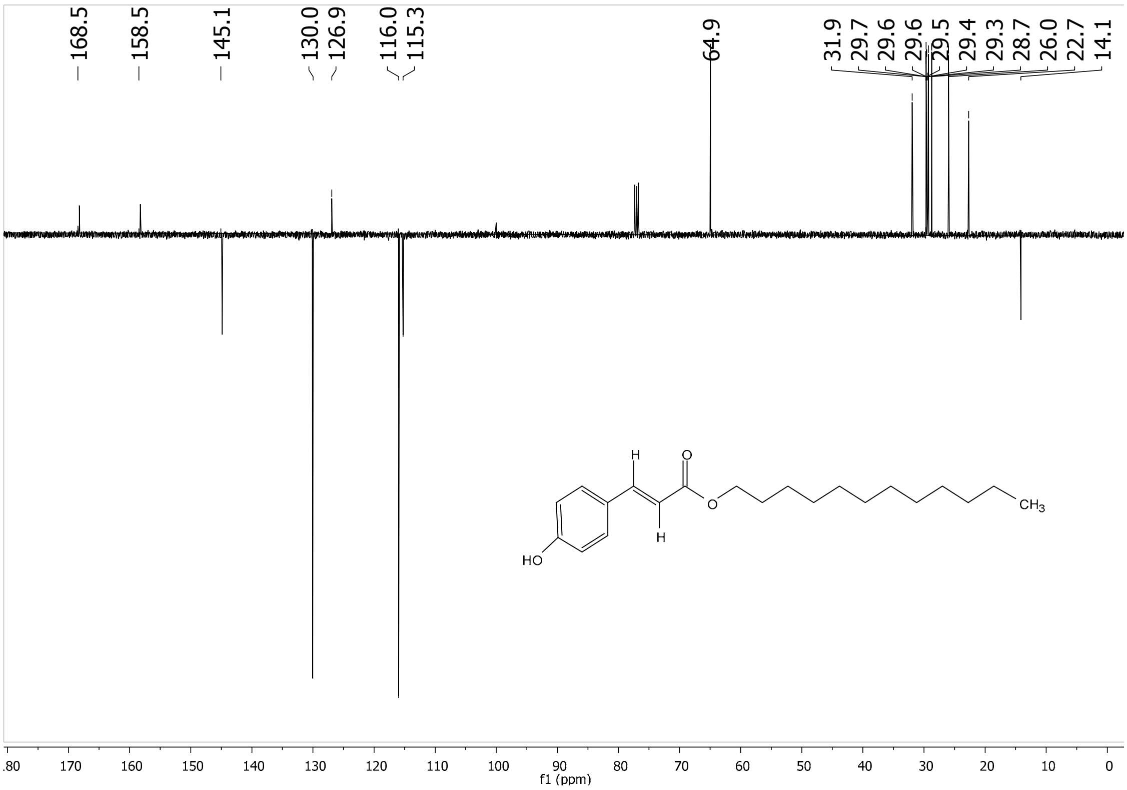

Figure S20. ^{13}C NMR (CdCl$_3$, 100 MHz) spectrum of dodecyl *p*-coumarate (**10**).

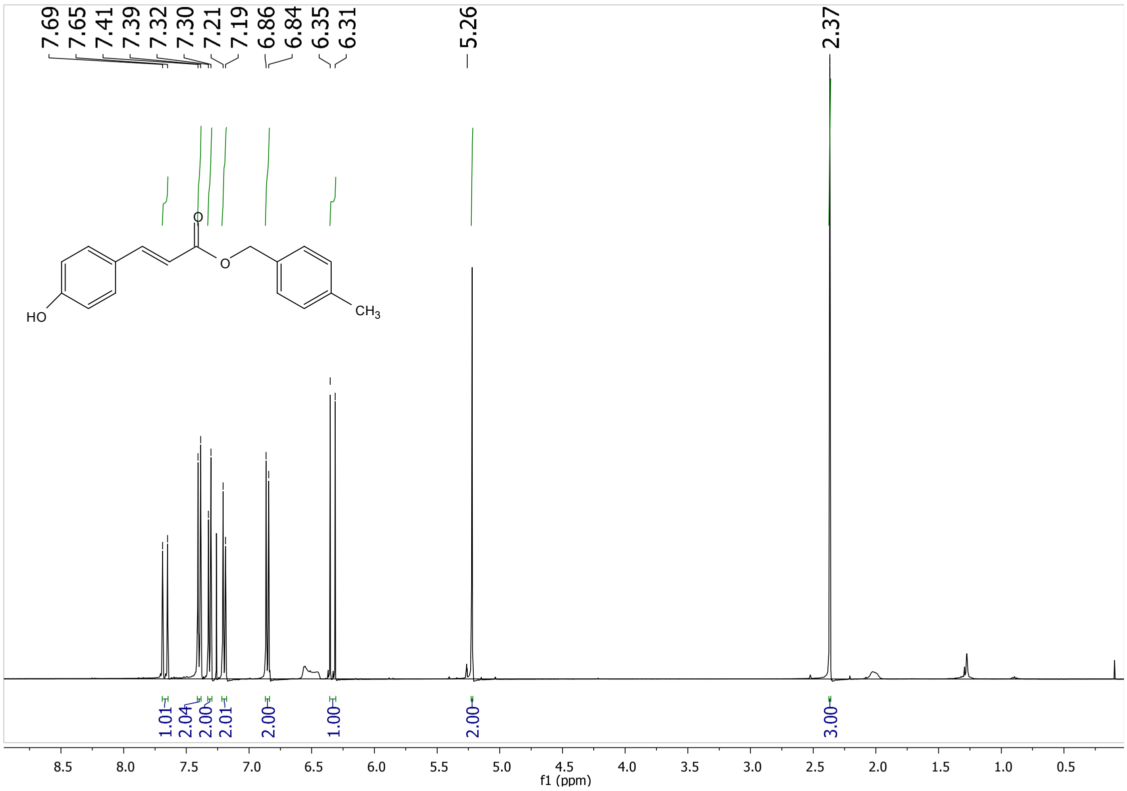

Figure S21. ^{1}H NMR (CdCl$_3$, 400 MHz) spectrum of 4-Methylbenzyl *p*-coumarate (**11**).

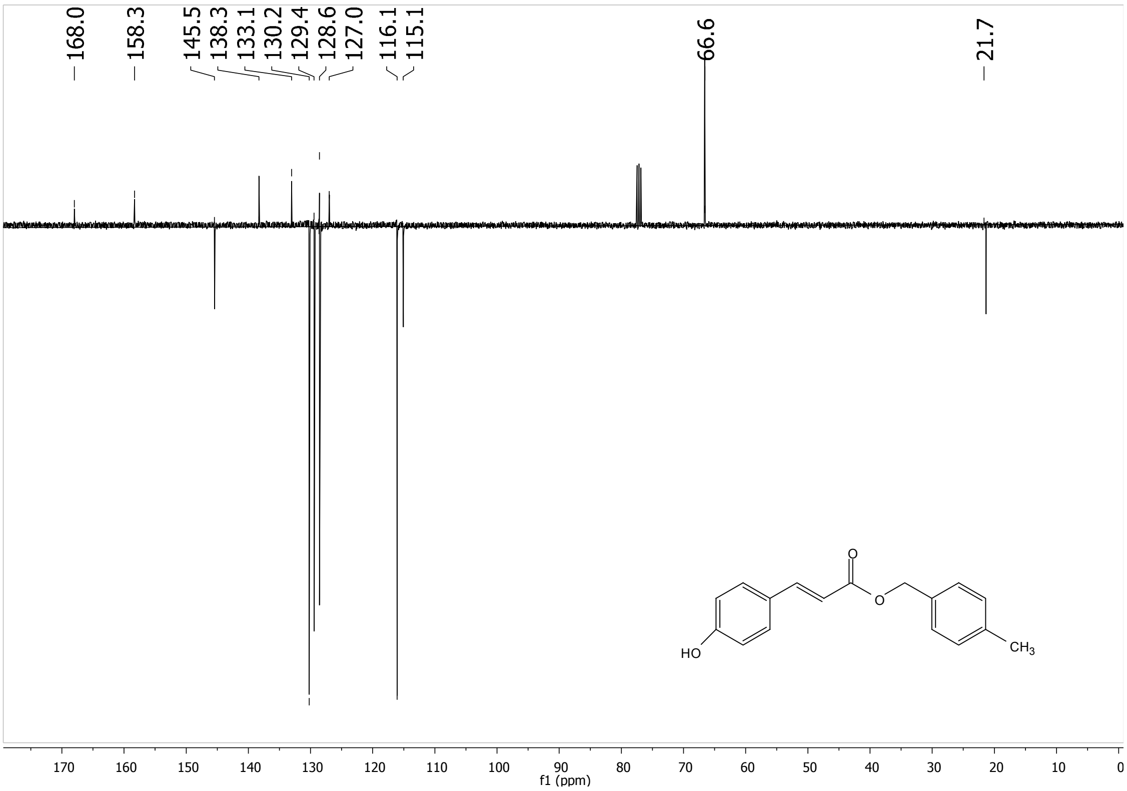

Figure S22. ^{13}C NMR (CdCl$_3$, 400 MHz) spectrum of 4-Methylbenzyl *p*-coumarate (**11**).

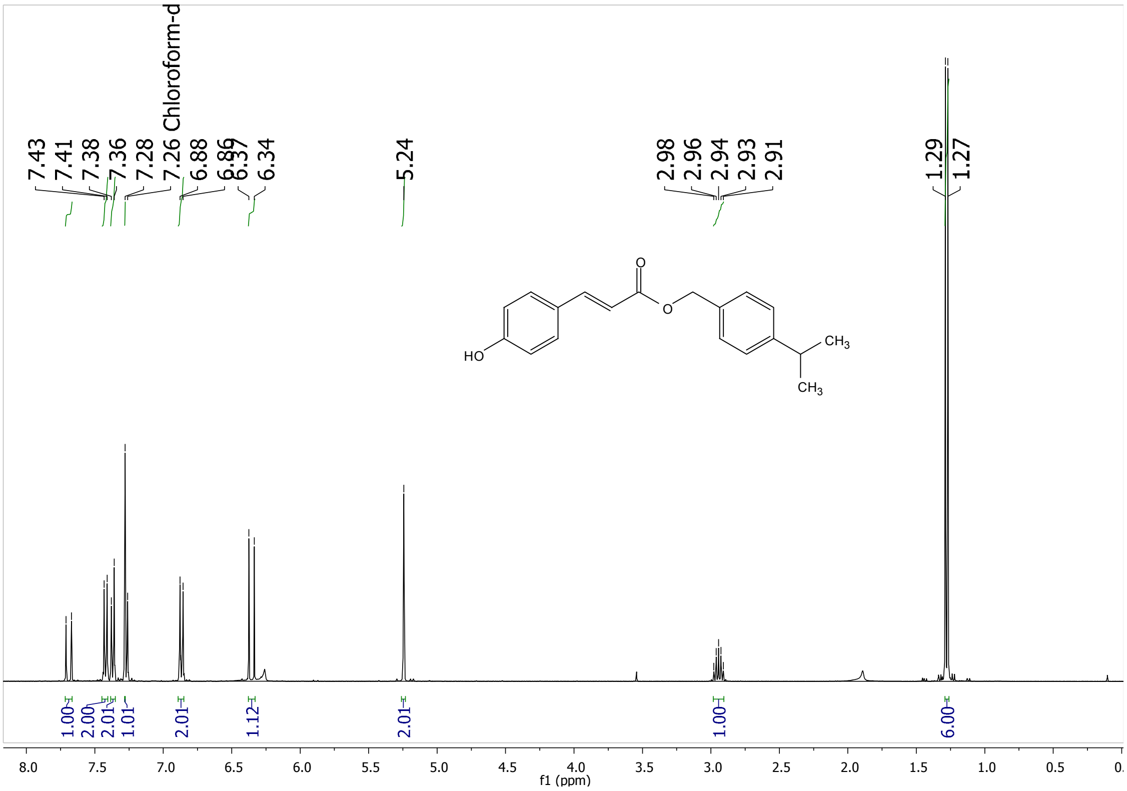

Figure S23. ^{1}H NMR (CdCl$_3$, 400 MHz) spectrum of 4-isopropylbenzyl *p*-coumarate (**12**).

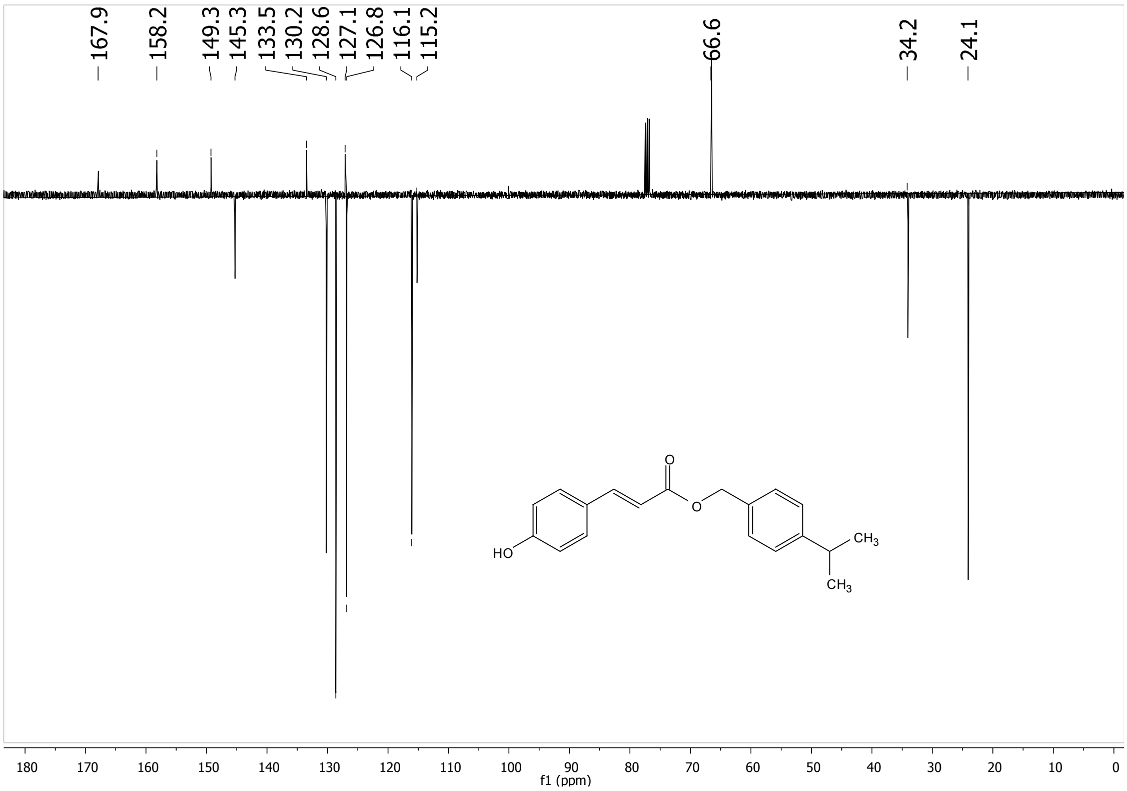

Figure S24. ^{13}C NMR ($CdCl_3$, 100 MHz) spectrum of 4-isopropylbenzyl *p*-coumarate (**12**).

References

1. Lopes, S.P.; Castillo, Y.P.; Monteiro, M.L.; Menezes, R.R.P.P.B.; Almeida, R.N.; Martins, A.M.C.; Sousa, D.P. Trypanocidal Mechanism of Action and in silico Studies of *p*-Coumaric Acid Derivatives. *Int. J. Mol. Sci.* **2019**, *20*, doi:10.3390/ijms20235916.
2. Khatkar, A.; Nanda, A.; Kumar, P.; Narasimhan, B. Synthesis, antimicrobial evaluation and QSAR studies of *p*-coumaric acid derivaties. *Arab. J. Chem*. **2017**, *10*, 3804–3815.
3. Allegretta, G.; Weidel E.; Empting M.; Hartmann R. W. Catechol-based substrates of chalcone synthase as a scaffold for novel inhibitors of PqsD. *Eur J Med Chem*. **2015**, *90*, 351–359.
4. Hixson, J.L.; Sleep, N.R.; Capone, D.L.; Elsey, G.M.; Curtin, C.D.; Sefton, M.A.; Taylor, D.K. Hydroxycinnamic Acid Ethyl Esters as Precursors to Ethylphenols in Wine. *J. Agric. Food Chem*. **2012**, *60*(9), 2293–2298.
5. Hosseini, R.; Moosavi, F.; Rajaian, H.; Silva, T.; Silva, D.M.; Soares, P.; Saso, L.; Edraki, N.; Miri, R.; Borges, F.; Firuzi, O. Discovery of neurotrophic agents based on hydroxycinnamic acid scaffold. *Chem Biol Drug Des*. **2016**, *88*(6), 926–937.
6. Nishimura, K.; Takenaka, Y.; Kishi, M.; Tanahashi, T.; Hiromi Yoshida, H.; Okuda, C.; Mizushin, Y. Synthesis and DNA Polymerase α and β Inhibitory Activity of Alkyl *p*-Coumarates and Related Compounds. *Chem Pharm Bull*. **2009**, *57*(5), 476–480.
7. Patel R.B.; Doshi, A. V. Synthesis and study of a new homologous series of Cis Cinnamate Esters of Mesogens: n-Hexyl-p-(p / -n-alkoxy cinnamoyloxy) Cinnamates. *Der Pharma Chemica*. **2011**, *3*(1), 338-348.
8. Kim, K.D.; Min, H.S.; Yum, E.K.; Ok, O.S.; Ju, Y.W.; Chang, M.S. Melanogenesis Inhibition by Mono-hydroxycinnamic Ester Derivatives in B16 Melanoma Cells. *Bull Korean Chem Soc*. **2010**, 31(1), 181–184.
